# Compositional analysis of bacterial peptidoglycan: insights from peptidoglycomics into structure and function

**DOI:** 10.1128/jb.00359-25

**Published:** 2025-10-09

**Authors:** Erin M. Anderson, Dyanne Brewer, Matthew T. Sorbara, Cezar M. Khursigara

**Affiliations:** 1 Department of Molecular and Cellular Biology, University of Guelph 317113, Guelph, Ontario, Canada; 2 Mass Spectrometry Facility, Advanced Analysis Centre, University of Guelph 3653, Guelph, Ontario, Canada; Geisel School of Medicine at Dartmouth, Hanover, New Hampshire, USA

**Keywords:** peptidoglycan, peptidoglycomics, bacterial cell wall, mass spectrometry, bioinformatic analysis

## Abstract

Peptidoglycan (PG) is a critical component of bacterial cell walls that stabilizes the cell membrane while performing diverse physiological roles. It consists of a polysaccharide backbone cross-linked by peptide side chains forming a lattice-like sacculus that encases the entire cell. The fundamental structural component known as a muropeptide is well characterized, although modifications to this structure are common and often linked to specific physiological functions. Recent advancements in mass spectrometry and bioinformatics now facilitate a detailed examination of the global composition of this essential biopolymer. We can deepen our understanding of its dynamic roles by employing peptidoglycomics to analyze how PG composition changes in response to physiological or environmental stimuli. This minireview will discuss the key physiological functions of peptidoglycan, introduce the peptidoglycomic approach, and highlight research where an omics-based perspective could significantly benefit future studies. By enabling a comprehensive, sensitive, and non-biased detection of PG modifications, peptidoglycomics provides a powerful lens to uncover novel structural variants and functional insights that were previously inaccessible using classical methodologies.

## INTRODUCTION

Peptidoglycan (PG) is a crucial biopolymer in the bacterial cell wall that has been the subject of intense study since it was first isolated in the early 1950s (1, 2). Over the last 70 years, extensive research has expanded our understanding of the structure and function of this microbial biopolymer. Recent advances in mass spectrometry and bioinformatics have revolutionized PG analysis, enabling a comprehensive detection of individual components and their global composition within bacterial cells. Like genomics, transcriptomics, and proteomics, peptidoglycomics is the non-targeted, non-biased detection of all elements that comprise the overall PG structure. Peptidoglycomic analyses can identify and monitor hundreds of potential compositional changes that occur within the PG structure of a cell. By comparison, traditional methods of analyzing PG composition only distinguish a relatively limited number of PG components. Therefore, peptidoglycomic approaches produce a detailed global overview of the PG structural elements and give unprecedented insight into the physiological function of this biopolymer within the bacterial cell.

This minireview will explore how peptidoglycan compositional analyses have advanced our understanding of PG by identifying modifications to its basic structure. It will also highlight several roles PG plays in biological processes and suggest areas of research where an 'omics'-based approach could be beneficial in uncovering how PG contributes to these functions. Through peptidoglycomics, we can gain valuable insights into how PG modifies in response to changes in bacterial physiology or environment.

## STRUCTURAL VARIABILITY OF MUROPEPTIDES IN GRAM-POSITIVE AND -NEGATIVE BACTERIA

**Fig 1:**
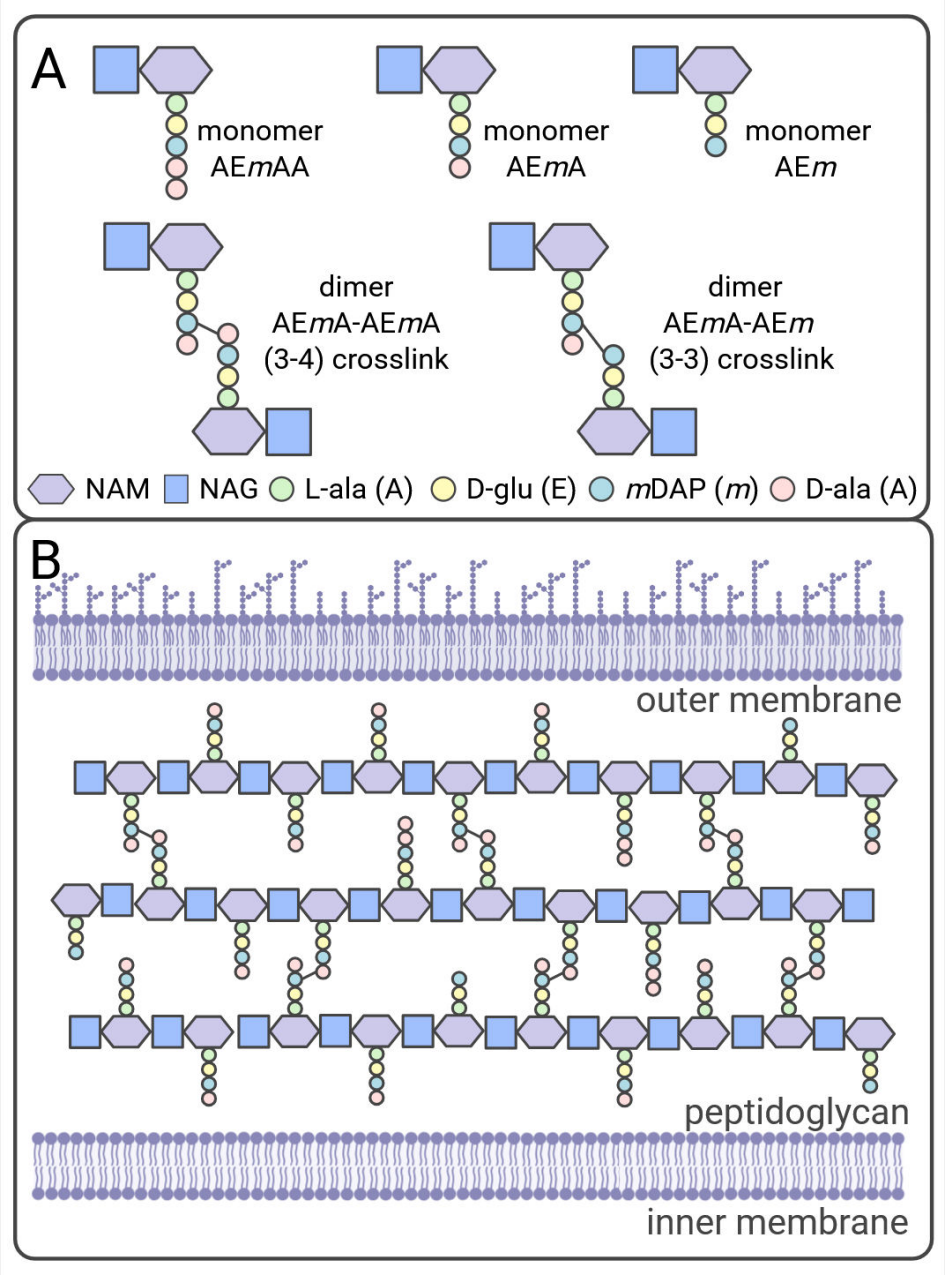
Basic peptidoglycan structure. (**A**) Illustrations of common muropeptide structures within gram-negative peptidoglycan. Muropeptides are named using the single amino acid code for the peptide side chain. (**B**) Representation of the mature peptidoglycan between the inner and outer membranes within gram-negative bacteria. Symbols for the NAG (blue square) and NAM (purple hexagon) are derived from the symbol nomenclature for glycans (18). Created with BioRender.com.

The basic subunit of the PG known as a muropeptide is a disaccharide of *N*-acetylglucosamine (NAG) and *N*-acetylmuramic acid (NAM) with a short peptide attached to the NAM (Fig. 1A). The muropeptide is β-1,4-linked to a glycan chain, and adjacent glycan strands are cross-linked via the peptide side chain to produce a lattice-like sacculus surrounding the entire cell (Fig. 1B). Bacteria can be classified as gram-positive or -negative based on differences in peptidoglycan structure and composition. Gram-positive bacterial cells have many cross-linked PG layers, 20 to 100 nm thick, that are external to the single-cell membrane (3–5). Gram-positive PG is interwoven with other glycopolymers, called teichoic acids and in some cases encapsulated by a layer of capsular polysaccharides and/or a protein layer called the S-layer (for a review, see [6, 7]). In comparison, gram-negative bacterial cells contain only a few layers of cross-linked PG, approximately 1–10 nm thick (8), in the periplasm between the inner and outer cell membranes (for a review, see [9, 10]). Mycobacteria have a very unique cell wall that shares aspects of both gram-positives and -negatives (for a review, see [11, 12]). The mycobacterial PG is thin, approximately 5–10 nm thick (13–15), and is covalently attached to a glycan of arabinose and galactose (arabinogalactan), which is, in turn, attached to mycolic acid, a part of the unique mycobacterial outer membrane. Additionally, the mycobacterial cell wall contains lipoglycans, such as lipoarabinomannan, that are attached to either the inner or outer membranes (16). The mycobacterial cell is further surrounded by a capsule consisting of mainly polysaccharides and proteins (12).

**Fig 2:**
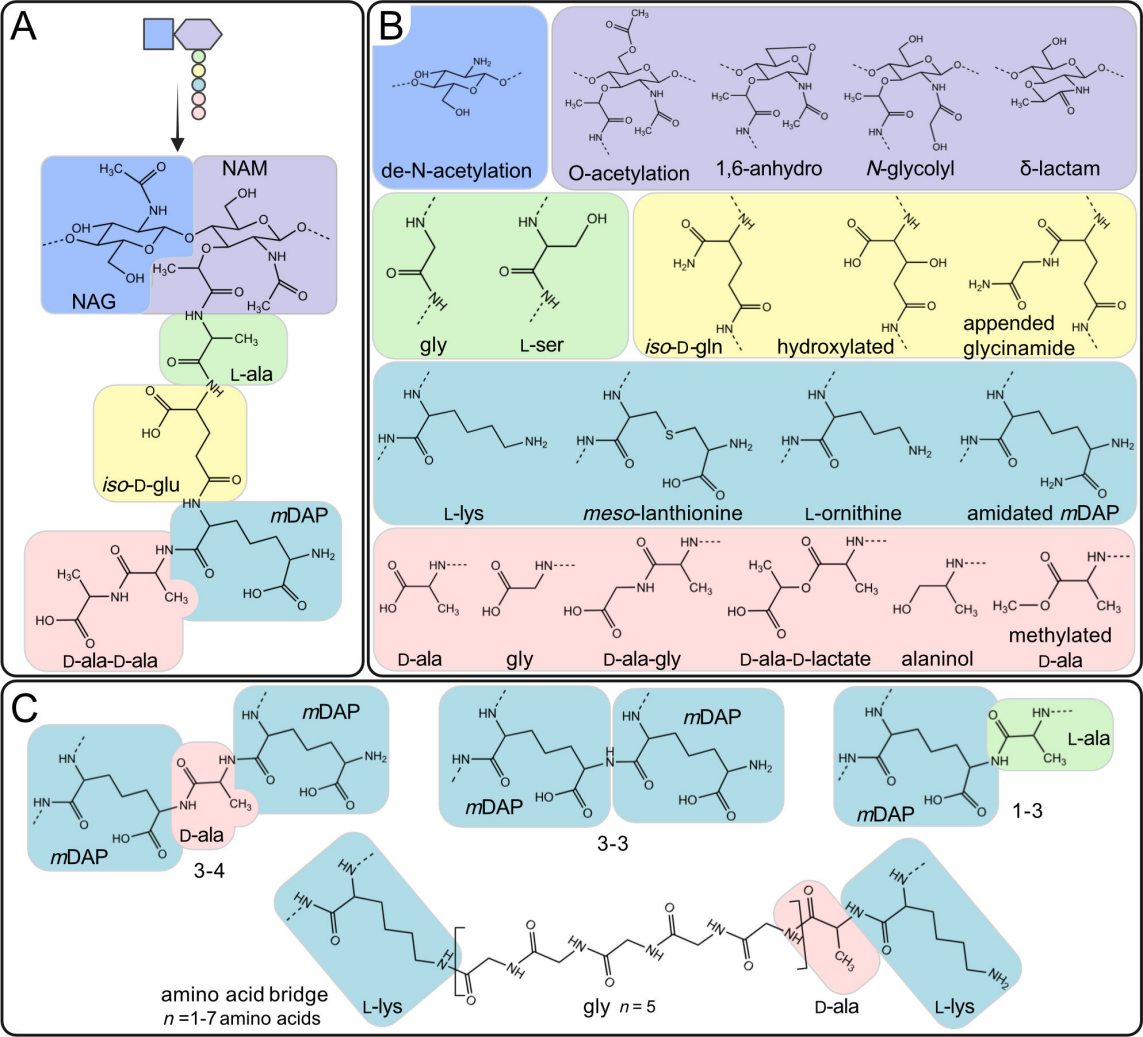
Muropeptide structures. (**A**) A cartoon illustration of the AE*m*AA muropeptide and the associated chemical structure with component sugars and amino acids color-coded. (**B**) Possible variations to the muropeptide chemical structure. Each moiety alternative can be superimposed on the corresponding similar colored position on the muropeptide structure in (**A**). When superimposed, the dashed lines indicate the bond of the following amino acid or sugar within the muropeptide structure. Additional modifications not listed are possible; for example, amino acids other than glycine can be substituted in either position 4 or 5 (pink). (**C**) Peptide crosslink variations between two adjacent muropeptides colored relative to the structure shown in (**A**). Created with BioRender.com.

Gram-negative bacteria have a basic muropeptide side chain with five amino acids consisting of ʟ-alanine, *iso*-ᴅ-glucosamine, *meso*-diaminopimelic acid (*m*DAP), ᴅ-alanine, and ᴅ-alanine appended to the NAG-NAM disaccharide via the ʟ-alanine (17) (Fig. 2A). For simplicity, the muropeptide side chain sequence can be written with the single letter amino acid code abbreviation, AE*m*AA, where the lower-case *m* represents *m*DAP (Fig. 1A). Adjacent stem peptides are crosslinked between the third amino acid, *m*DAP, on one stem peptide and the fourth amino acid, ᴅ-alanine, on the second stem peptide, with the additional loss of the terminal ᴅ-alanine creating AE*m*A. This is also referred to as a 3–4 crosslink (Fig. 2C). The 3–4 crosslink is the most abundant covalent bond between muropeptides, although linkages between other amino acids in the peptide side chain are possible (Fig. 2C).

In gram-positive bacteria, the composition of the PG can vary considerably between different species. A review by Schumann (2011) contains a list of variations in gram-positive muropeptide structures (19). One common component that differs is the presence of a short interpeptide crossbridge consisting of one to seven amino acid residues (20) that links between the third (most often not *m*DAP) and fourth amino acids of two adjacent peptide side chains (Fig. 2C). For mycobacteria, although part of the gram-positive phylum *Actinobacteria*, muropeptides contain *m*DAP and no interpeptide crossbridge (11). However, mycobacterial muropeptides often contain a unique *N*-glycolyl NAM modification (11, 21)(Fig. 2, purple).

Among gram-negative and -positive organisms, there can be a considerable number of modifications to these basic muropeptide structures, with variations in the stem peptide composition, sugar and/or peptide modifications, and variations in types of stem peptide crosslinks (Fig. 2B and C). Table S1 provides an extensive list of species that modify their PG muropeptide structure, including the known genes responsible for this modification, as well as the associated physiological processes.

## EVOLVING METHODOLOGIES IN PEPTIDOGLYCAN ANALYSIS

**Fig 3:**
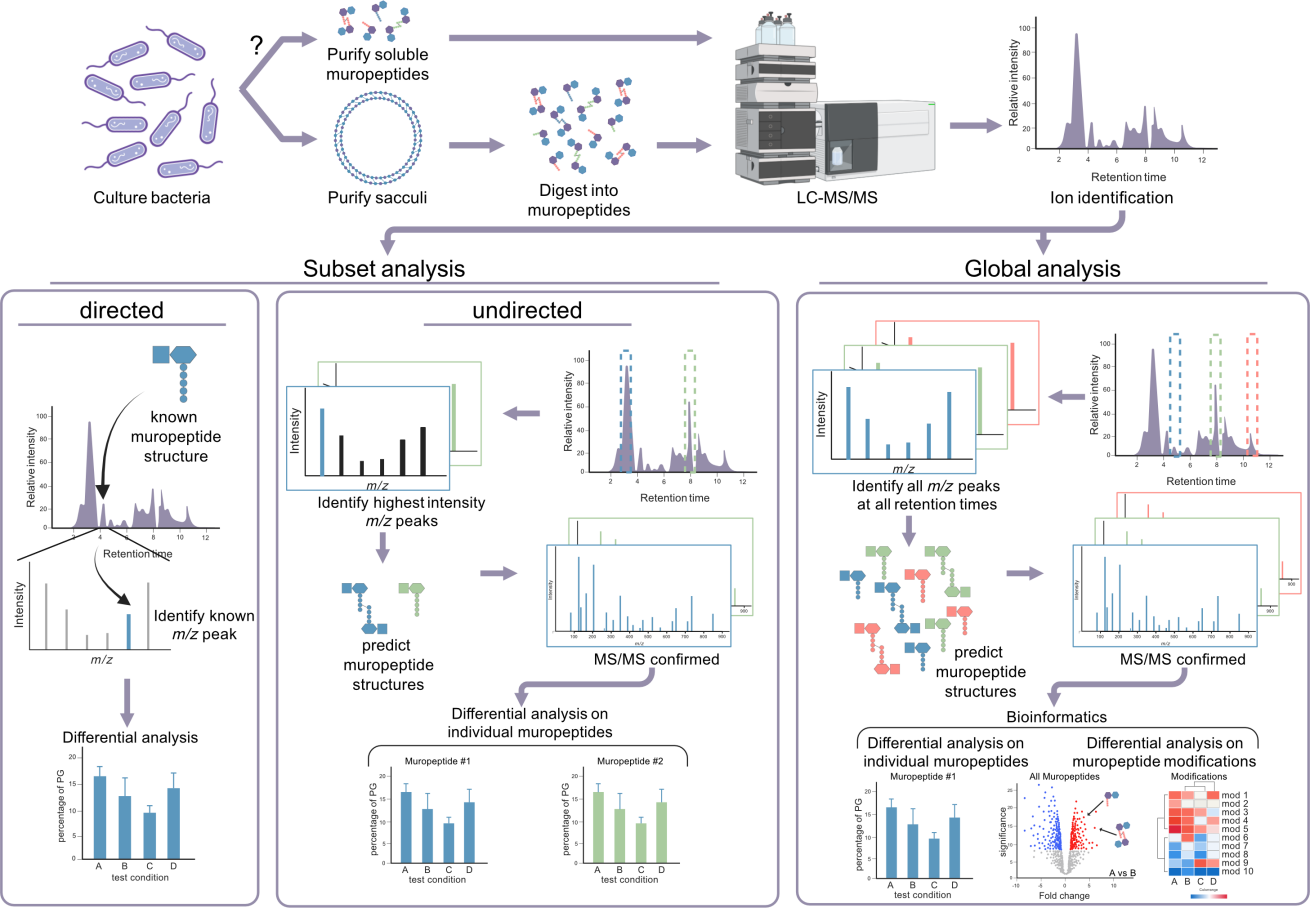
Comparison of the peptidoglycan compositional analysis techniques. A typical peptidoglycan compositional analysis workflow entails the culture of bacteria, followed by either peptidoglycan sacculi purification, typically extracted by boiling in sodium dodecyl sulfate or isolation of the soluble muropeptides from the culture media. A method for purifying soluble muropeptides that is compatible with peptidoglycomic analysis has not yet been tested. Sacculi are digested into the individual muropeptides through the activity of a muramidase prior to analysis. Reverse-phase liquid chromatography is used to separate muropeptides, followed by analysis by mass spectrometry. Mass spectrometer ion chromatograms can be analyzed following two distinct methodologies. With the directed subset approach, chromatograms are inspected for known *m/z* peaks representing known muropeptide structures, and the relative intensities of these known structures are compared across samples. The undirected subset approach inspects chromatograms for the highest abundance peaks. These peaks are identified for the representative muropeptide structure, and the relative intensities of these individual structures are compared across samples. With a global approach, all *m/z* peaks representing all the muropeptides comprising the global peptidoglycan composition are identified within each sample using feature extraction algorithms. Each *m/z* peak at each retention time is annotated for the representative muropeptide structure and confirmed with MS/MS. Using bioinformatic approaches, these large data sets can be analyzed for global changes and trends of the PG population and the relative intensity of the individual muropeptides or muropeptide modifications across samples. Created with BioRender.com.

The first studies on PG composition involved breaking the sacculi down into individual amino acid and sugar components and using methods, such as two-dimensional thin-layer chromatography, to identify the elements (2). Over the years, studies on the PG composition have led to numerous technological improvements, increasing detail and accuracy while reducing the labor required. Current peptidoglycomics methodology, to some extent, resembles bottom–up proteomics (22), in that PG sacculi are broken down into easier-to-analyze ‘fragments’ (i.e., muropeptides) using a muramidase to digest the sugar backbone. Muropeptides are then separated and identified using high-resolution reverse-phase liquid chromatography (LC) that is coupled to high-powered tandem mass spectrometry (MS) (Fig. 3). Over the last few years, advances in MS technology have significantly increased detection sensitivities, thereby enhancing the ability to identify the muropeptide composition of a sample in detail. Bioinformatic MS feature finding and statistical data analysis software processes and analyzes these complex MS data sets to identify the compositional changes in the context of the PG structure. Overall, both classical and current methodologies for the peptidoglycan compositional analyses can be separated into two distinct strategies: either a subset or a global analysis.

### Subset analysis

Classical peptidoglycan compositional analyses represent a subset approach, targeting only part of the total muropeptide population, and can be further categorized as directed or undirected (Fig. 3). The muropeptides identified in this approach can represent specific modifications the researchers are targeting for analysis (directed) or an overall unbiased scan of the most abundant moieties within the overall PG structure (undirected). Investigating a directed subset of known muropeptides is a hypothesis-driven approach that requires previous knowledge of predicted structural changes within the PG. The MS data are assessed for the presence or absence of known mass-to-charge (*m*/*z*) peak(s) that represent a specific muropeptide or group of muropeptides (Fig. 3). Given that 70+ years of research have produced extensive knowledge of peptidoglycan structure and possible modifications (e.g., Table S1), many studies employ a directed subset approach to focus on specific, well-characterized features. For instance, a directed subset approach was successfully applied to identify the unique L,D-transpeptidase responsible for 1–3 crosslinks in *Alpha*- and *Betaproteobacteria*. These modifications are thought to enhance cell wall resilience under specific environmental stresses (23).

In contrast, an undirected subset compositional analysis (Fig. 3) employs a non-biased approach to identify the most abundant muropeptides within the sacculi. Historically, classical PG compositional studies required labor-intensive methods, such as manually isolating individual muropeptides using HPLC prior to MS detection. This challenging and time-consuming process limited the number of muropeptides identified, as these analyses typically focused on the most abundant and, therefore, most easily purified muropeptides. For instance, Glauner et al. (17) identified approximately 80 distinct muropeptides in *Escherichia coli*, whereas Lee et al. (24) identified only 20 distinct muropeptides in *Pseudomonas aeruginosa* (24). Despite these limitations, early studies laid the foundation for our understanding of the peptidoglycan compositional variability, revealing the basic structure of gram-negative PG, the diversity of gram-positive PG structures, and various PG modifications.

### Global analysis

The global PG compositional approach, a hallmark of peptidoglycomics, relies on advanced LC-coupled MS technologies with high sensitivity to detect the full spectrum of muropeptides within the cell wall (Fig. 3). This technique assesses all the measurable muropeptides within the PG structure and provides a more comprehensive assessment of the overall PG composition. For example, our group identified several hundred distinct muropeptides within the entire global PG composition of *P. aeruginosa* (25) compared to 20 muropeptides in the classical subset analysis of the same species (24). The increased sensitivity of our methodology enabled the identification of numerous muropeptides that were not identified in the earlier study, including the de-N-acetylation of NAG (Fig. 2B, blue) (25). This peptidoglycomic methodology is highly sensitive and can detect as few as 300–400 muropeptides per cell with a particular modification (25). Within a peptidoglycomic global analysis, the application of advanced MS feature extraction enables the automated identification and monitoring of thousands of mass-to-charge ratio (*m*/*z*) peaks simultaneously. This includes all various charges, adducts, isotopes, and common in-column degradation products (e.g*.,* loss of NAG) that can result. A more robust and accurate abundance assessment is obtained by identifying and pooling all *m*/*z* ions representing a single muropeptide structure.

The increased sensitivity of a global peptidoglycomics approach should prove helpful in identifying unique muropeptide modifications that are less abundant in the overall global PG structure. For instance, a recent global analysis of the PG composition of the pathogen *Salmonella enterica* serovar *Typhimurium* persisting inside fibroblasts contains a unique muropeptide consisting of an amino alcohol (alaninol) replacing the terminal ᴅ-alanine of the stem peptide (26) (Fig. 2B, pink). This unique muropeptide modification was only found when the pathogen grew intracellularly and may be linked to immune evasion inside host cells. This modification comprised just 1.5% of the total PG content of the intracellularly grown *S*. *typhimurium*, a relatively small portion of the overall PG structure of the cell. The PG of *E. coli* has been estimated to contain approximately 3.5 × 10^6^ muropeptides per cell (27). Thus, 1.5% could amount to ~50,000 copies of this specific modification per cell, suggesting a biologically relevant effect on the overall physiology of the bacterial cell.

Recently, our group described two subsets of muropeptides that varied in overall abundance (high vs. low abundance) per cell and carried out distinct physiological roles within *P. aeruginosa* PG (25, 28). The most abundant muropeptides represented those with minimal alterations from the standard AE*m*AA configuration and were referred to as ‘core’ muropeptides. The composition of this core PG was relatively consistent, regardless of strain or growth condition (25, 28), and may serve as a structural framework for the overall PG sacculi. Given that the most abundant muropeptides are typically identified using an undirected subset analysis, this strategy can also be considered a practical method for profiling the core PG composition. The second subset, the ‘adaptive’ muropeptides, was observed in lower abundance but exhibited a significant variability between strains or growth conditions (25, 28). This adaptive subset of muropeptides could allow flexibility in response to physiological stimuli, while the lower abundance would not affect the overall structural integrity. The increased sensitivity provided by the peptidoglycomic global analysis approach will be indispensable for determining the physiological roles of these lower-abundant adaptive muropeptides.

## CHALLENGES AND STRATEGIES TO CONSIDER WITH PEPTIDOGLYCOMIC ANALYSIS

**Fig 4:**
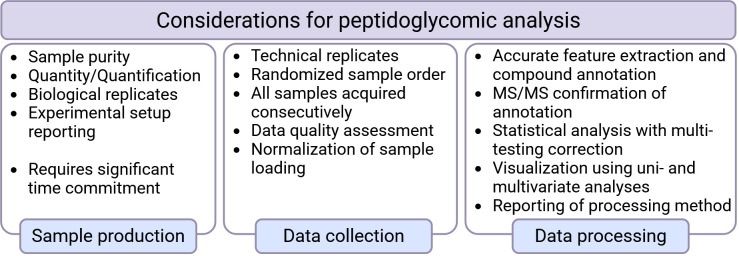
Points to consider when performing peptidoglycomic analysis. Created with BioRender.com.

This global peptidoglycomics approach has several advantages over more traditional techniques, including the sensitivity and detailed information provided in the resulting data. However, several parameters must be considered to maintain the robustness and validity of the results (Fig. 4). For example, peptidoglycomic studies should adopt a minimal reporting guideline, similar to reporting guidelines established for proteomic (29) and metabolomic (30, 31) workflows. These guidelines would provide the details required for reporting experimental setup and data processing to ensure clarity and data quality for downstream research use. Additionally, due to the difficulty and time commitment needed for preparing PG sacculi, many PG compositional studies have employed minimal technical replicates (i.e*.*, multiple MS runs) of a single biological replicate. For these complex global peptidoglycomic assessments, biological and technical replicates are required to perform advanced bioinformatic data analyses. In addition, as the sacculi purification protocol remains cumbersome and very time-consuming, partial loss of the sample is possible. Therefore, normalization of sample loading is critical. This ensures that technical variations do not influence muropeptide abundance during MS analysis. Various techniques can be employed for normalization, including colorimetric (diamino detection [32, 33] and Nelson-Somogyi [34]) and reduced sugar detection with either high-performance liquid chromatography (HPLC) with pulsed-amperometric electrochemical detection (35) or MS detection (capillary electrophoresis-coupled [36] or derivatized [37, 38]). Lastly, minute changes in the responsiveness of the MS-instrumentation over time can result in shifts in ion intensities and retention times. Therefore, samples for an entire experiment should have a randomized sample order and be analyzed consecutively.

One of the most critical aspects of mass spectrometry analysis is the accurate extraction of features and the annotation of compounds. Currently, mass spectrometry peak annotation software is designed for either proteomic or metabolomic analyses, and neither is built to thoroughly analyze the unique parameters of the PG sugar/peptide structure. However, several dedicated peptidoglycomic programs have been developed over the last few years. PGFinder (39–41) compares *m*/*z* peaks against a built-in or a user-built muropeptide library. Once muropeptide structures are predicted, MS/MS data can be used for the manual confirmation of the structure. MS/MS spectral libraries are often used for automated compound identification in proteomic or metabolomic data sets. However, muropeptides consist of both sugar and peptide moieties. Therefore, current programs built for other ‘omic’ data sets do not accurately identify PG muropeptides. Recently, two programs for the *in-silico* prediction of MS/MS muropeptide fragmentation have been developed to annotate peptidoglycomic data sets autonomously. The PGN_MS2 (42) and the high-throughput automated muropeptide analysis (HAMA) (43) programs are freely available and identify known muropeptide structures for gram-positives and -negatives using predicted MS/MS fragmentation. However, there are still limitations with either program. This includes a requirement for previous knowledge of likely PG composition and limitations on the muropeptide modifications or crosslinks included within the library generation. Each requires additional programs for robust feature extraction. However, these programs represent a good step toward a dedicated, fully automated muropeptide prediction program.

As with other ‘omic’ data analysis pipelines, using bioinformatic computational tools to manage and evaluate these relatively large data sets is advantageous (Fig. 3). These tools can include various quality control measures, as well as univariate and multivariate analysis methods. Univariate methods examine each *m*/*z* feature (i.e., muropeptides) independently and apply statistics, such as *t*-tests, to separately compare the abundance changes of each muropeptide between different samples. Due to the number of features examined, these statistical tests are repeated many times. Therefore, multiple testing corrections, such as the Benjamini-Hochberg false discovery rate, should be applied to avoid false positive results (44). Multivariate analyses are pattern-recognition methods that simultaneously examine all the *m*/*z* features within the data set to describe global correlations within the data. One such multivariate analysis, the principal components analysis (PCA), indicates the sample/data quality and large-scale trends in the data, as well as potentially identifying sample outliers. Using these advanced bioinformatic analyses, such as PCA, hierarchical clustering, volcano plots, and others, is a powerful way to visualize relatively large data sets to identify trends and highlight unique features (for a review of these computational tools, see [45]). These visualization methods enable the identification and tracking of all muropeptides within the global PG structure (Fig. 3), making the interpretation of these complex compositional data sets easier. For example, entire groups of muropeptides sharing the same modification can be assessed for significant changes in abundance. Monitoring a specific modification across multiple muropeptides can provide strong evidence that the modification plays a critical role under the tested conditions. Additionally, in instances where the abundance of a muropeptide in a test condition deviates from the trend of others with the same modification, it may suggest the involvement of an enzyme with unique specificity or a distinct biological function.

## AVENUES FOR EXPLORATION: STUDYING BACTERIAL PHYSIOLOGY, ANTIMICROBIAL RESISTANCE, AND HOST INTERACTIONS USING PEPTIDOGLYCOMICS

**Fig 5:**
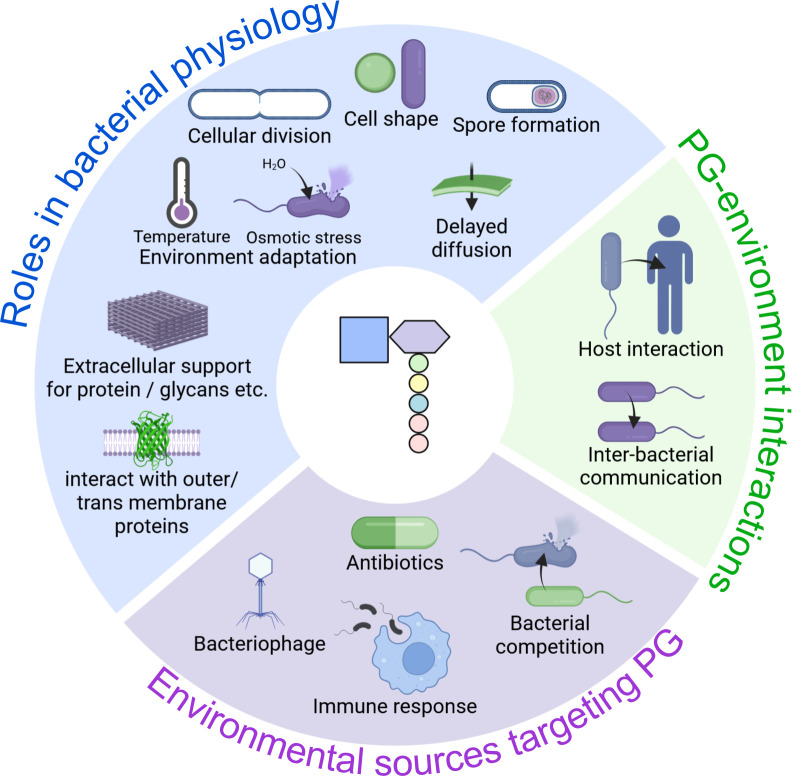
Overview of the physiological roles and environmental interactions involving bacterial peptidoglycan (PG). Peptidoglycan plays a central role in bacterial physiology (blue), including maintaining cell shape, mediating cell division, enabling spore formation, supporting extracellular protein and glycan structures, mediating protein interactions, and adapting to environmental stresses, such as osmotic pressure and temperature. PG also serves as a key interface for bacterial interactions with the environment (green), including host-pathogen interactions and inter-bacterial communication. Conversely, PG is targeted by various environmental sources (purple), such as antibiotics, bacteriophages, bacterial competitors, and the host immune system. These selective pressures contribute to the evolution and functional diversification of PG structure and composition. Created with BioRender.com.

Decades of research on peptidoglycan have revealed its highly complex physiological roles, many of which are beyond the scope of this minireview, such as involvement in cellular division (46, 47), cell shape determination (48, 49), and spore formation (50, 51), among others (Fig. 5). Thus, this section will focus on a few essential physiological roles of PG related to antimicrobial resistance and host interactions and highlight areas where the application of peptidoglycomics may be beneficial (Fig. 3).

### Physiological role of unique PG modifications

To date, many muropeptide modifications have been identified. Some muropeptide modifications predominate in specific bacterial species, such as the amidated *m*DAP (Fig. 2B, teal), which are found in high concentrations in *B. subtilis* (52). Other muropeptide modifications are found in relatively low abundance within the overall PG composition. In some cases, bacteria contain dedicated enzymes for producing a specific muropeptide modification (Table S1). For example, the GatD/MurT complex within the cytosol of gram-positive cells adds an amine to the α-carboxyl group of ᴅ-*iso*-glutamate in the stem peptide, producing ᴅ-*iso*-glutamine (Fig. 2B, yellow) (53, 54). In other cases, variability in the PG composition could result from enzymes possessing less restrictive specificity, which creates a ‘leaky’ incorporation of non-canonical moieties. For example, the cytosolic enzyme MurE typically involved in incorporating the third amino acid (*m*DAP) into the stem peptide in gram-negative bacteria can also add other amino acids to the muropeptide structure but at a lower efficiency (55, 56). As *m*DAP is recognized by NOD1 (57), the leaky incorporation of different amino acids could have implications for bacterial-host interactions. In our global peptidoglycomics analysis of *P. aeruginosa*, distinct muropeptides were identified, which could indicate the activity of a leaky MurE enzyme (25). Other non-canonical variations of the stem peptide could occur from species-distinctive changes in enzyme specificity, such as glycine or serine replacing the ʟ-alanine (Fig. 2B, green) of the stem peptide via MurC activity (58, 59).

Of the many known muropeptide modifications, most have been associated with some form of physiological function (Table S1). For example, a muramic δ-lactam (Fig. 2B, purple) on the glycan backbone is essential in *B. subtilis* and *Clostridium difficile* spore production (51, 60). An N-glycosyl modification of the NAM residue (Fig. 2B, purple) influences the host-mycobacterial immune responses (21). The presence of a glutamine in the second position of the stem peptide influences cross-linking abundance in the gram-positive *Streptococcus pneumoniae* (61). However, the function of other modifications remains to be elucidated, such as the methylation on either the glutamate, *m*DAP, or the terminal alanine found in *Mycobacterium smegmatis* (62) or the methylated terminal alanine in *P. aeruginosa* (25) (Fig. 2B, yellow, teal, and pink, respectively).

As the highly sensitive peptidoglycomics approach is applied across more bacterial species and growth conditions, additional unique muropeptide modifications are likely to be discovered, such as the unique alaninol modification found in intracellularly growing *S*. *typhimurium* (26). As additional nuances in PG composition are uncovered, our understanding of the physiological roles of this essential bacterial biopolymer will continue to deepen.

### Multiplicity of periplasmic enzymes involved in peptidoglycan synthesis and modification

**Fig 6:**
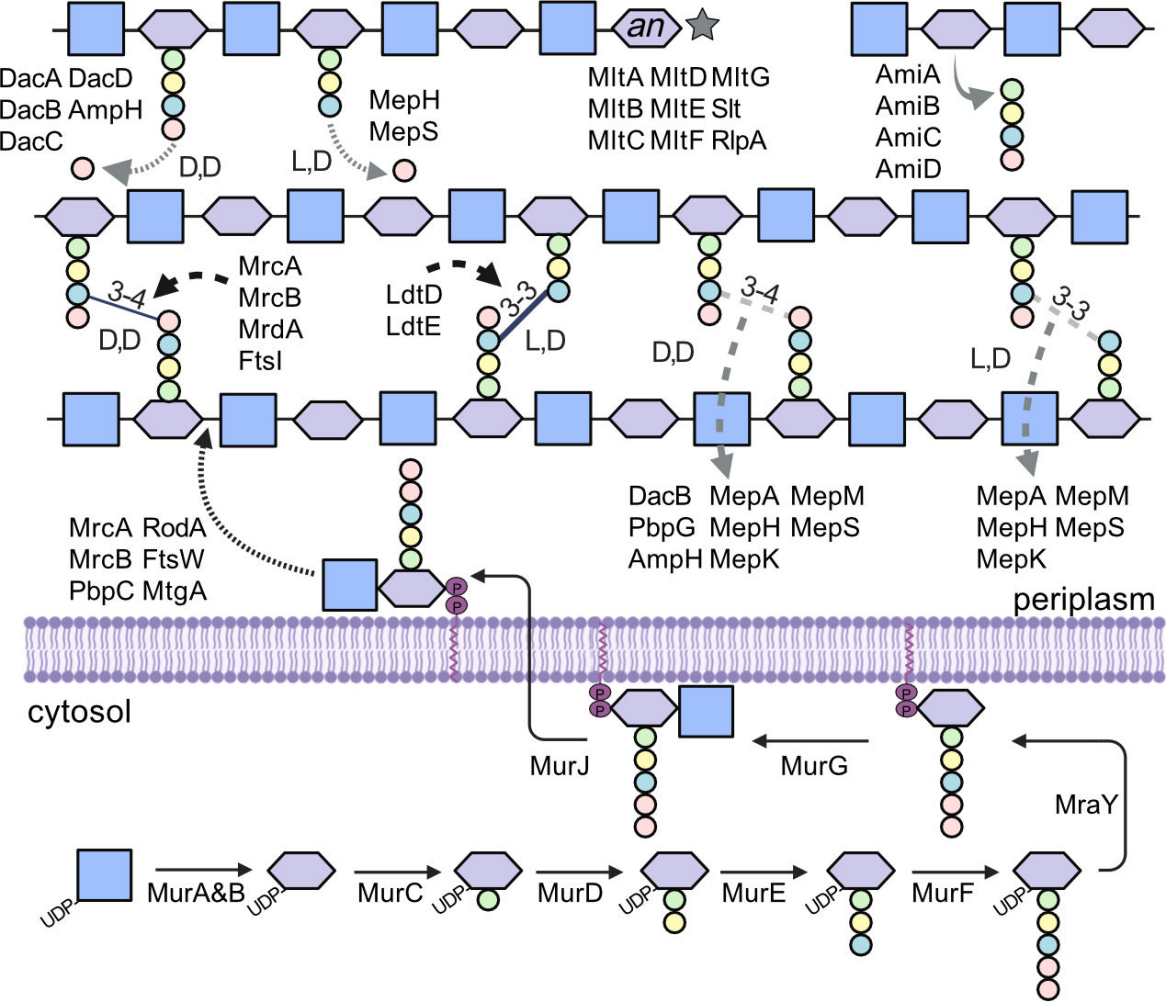
Peptidoglycan synthesis and hydrolase pathway in *E. coli*. (Black arrows) PG synthesis. A series of dedicated enzymes (solid black arrows) located within the cytosol successively add the individual components to produce the fundamental muropeptide precursor. This muropeptide precursor is bound to the inner leaflet of the inner membrane by undecaprenol phosphate before being flipped to the outer leaflet. Once on the periplasmic side of the inner membrane, the muropeptide precursors are added to the mature peptidoglycan structure through the activity of multiple transglycosylases (short dashed black arrows) and transpeptidases (long dashed black arrows). (Gray symbols) PG hydrolases. Within the periplasm, numerous hydrolases are responsible for the degradation of the mature PG structure to make space within the rigid lattice for new PG synthesis and/or transmembrane protein complexes. These hydrolases include amidases (solid gray arrows), carboxypeptidases (short dashed gray arrows), endopeptidases (long dashed gray arrows), and lytic transglycosylases (gray stars). Sugar/peptide symbols represent (blue square) NAG, (purple hexagon) NAM, (purple hexagon with *an*) 1,6 anhydro NAM, (circles) amino acids color-coded to Fig. 2, (green) ʟ-alanine, (yellow) *iso*-ᴅ-glutamate, (teal) *m*DAP, and (pink) ᴅ-alanine. Created with BioRender.com.

Due to the importance of the PG structure on cell viability, the production, modification, and degradation of PG are highly regulated. PG synthesis has been well-studied and begins with precursor production within the cytosol, followed by the final PG structure’s assembly in the periplasm (Fig. 6) (for a review, see [9, 63–65]). For the PG assembly to occur, space in the lattice-like mature PG structure must first be generated via the cleaving activity of PG-specific hydrolases (reviewed in [66]). These hydrolases include enzymes that cleave the peptide side chain (amidase, endopeptidase, carboxypeptidase), as well as enzymes that cleave the glycan strand (muramidase, glucosaminidase, lytic transglycosylase). Once space is produced, the PG synthase enzymes can add new muropeptide subunits via transglycosylase and transpeptidase activity. The enzymatic activity acting on the mature PG structure within the periplasm, including both the hydrolase and synthase enzymes, is carried out by multiple enzymes capable of performing the same enzymatic reaction (Fig. 6). For example, several lytic transglycosylases degrade the polysaccharide backbone during PG recycling by cleaving the NAM-β−1,4-NAG bond, producing a glycan chain terminus with a 1,6-anhydro (anh) ring on the NAM (for a review, see [67]) (Fig. 2B, purple, Fig. 6). In total, six families of lytic transglycosylases have been identified, and most bacteria contain multiple versions of these enzymes across several of these families. For instance, *E. coli* encodes nine, whereas *P. aeruginosa* encodes 11 lytic transglycosylases (Table S1), yet all perform the same enzymatic reaction (67).

It has been proposed that the appearance of redundancy in the PG enzymes outside of the cytosol is in response to the variable conditions present in the microenvironment surrounding the PG (either in the periplasm for gram-negative or in the external environment for gram-positive) in comparison to the more regulated and buffered environment within the cytosol (65). Indeed, some of these seemingly redundant enzymes have varied activities under different environmental conditions. In *E. coli*, the lytic transglycosylases MltE and MltC are more active at acidic pH (68). Likewise, the PG synthesis enzyme complexes exhibit similar pH sensitivity, with the MrcA/LpoA complex being the most active under alkaline conditions and the MrcB/LpoB complex showing the most activity in acidic conditions (69) (69). Osmolarity can also play a role; for example, in *Vibrio cholerae*, an endopeptidase was essential for viability only when salt was present (70). In addition to varied activities under distinct environmental conditions, the multiplicity of these enzymes can also result from the localization of enzymatic activity to specific regions in the cell. For example, two PG synthase complexes are functionally separated within the cell. The complex containing MrdA/RodA is responsible for PG synthesis of the lateral cell wall. In contrast, the FtsI/FtsW complex is responsible for PG synthesis at the cell poles during cell division (10, 71). One mostly overlooked aspect of PG-acting enzymes is that they function on a mature PG structure. Therefore, in addition to regional functionality or increased activity due to environmental conditions, the localized PG composition could influence these similar PG enzymes. Recently, Razew et al. demonstrated that two isoenzymes purified from *Staphylococcus aureus* catalyzed the same crosslink hydrolysis (72). However, only one was capable of efficient activity in a highly crosslinked PG structure, such as in the mature sacculi. This demonstrates that although two enzymes may appear to have redundant functions *in vitro*, they may have distinct specificities within the context of the whole structure.

In the future, using a highly sensitive peptidoglycomic approach may help clarify individual PG-related enzyme specificities. For example, examining the abundance differences of individual muropeptides within the group of muropeptides containing a specific modification in detail may suggest where enzyme specificity requires the presence of additional modifications for functionality. For instance*,* muropeptides with a second-position amine influence the ability to crosslink between muropeptides in some species (61). Further work will be needed to determine whether the multiplicity of the PG enzymes is due to localized regional activity in the cell, the effect of variable environmental conditions, or caused by localized specificity to the overall PG structure. A prerequisite for disentangling these functions is the ability to analyze the global PG structure with sufficient sensitivity using the peptidoglycomic methodology.

### Physiological role of peptidoglycan during antimicrobial resistance

Due to the importance of PG integrity for bacterial survival, antimicrobials that target PG synthesis pathways are widely used in medicine (63). For example, β-lactam antibiotics block the active site of PG synthesis enzymes, which disrupts the production of new PG. When PG synthesis is disrupted, the overall structure of the cell membrane is weakened, resulting in increased susceptibility to cell lysis. Early studies investigating PG composition led to the discovery of a specific modification to PG muropeptides that consists of a crosslink between the third amino acids of two adjacent peptide side chains (Fig. 2C) (73). These 3–3 crosslinks allow the PG structure to be maintained during cell envelope stress, such as when PG synthesis enzymes are inhibited by β-lactam antibiotics (for a review, see [74, 75]). Moving forward, the high sensitivity of peptidoglycomics methodology will likely reveal further unique muropeptide modifications or crosslinks occurring during antimicrobial treatments, indicating potential unique enzymes involved during resistance. These could uncover additional mechanisms of antimicrobial resistance and provide deeper insights into the role of PG in the increasingly critical battle against antimicrobial resistance, such as the recently described novel crosslink between the first and third amino acids (1–3) of adjacent muropeptide side chains (Fig. 2C), which may represent another mechanism to strengthen the cell wall during stress (23, 76, 77).

### Role of peptidoglycan during host-pathogen interactions

Peptidoglycan muropeptides are critical signaling molecules in the extracellular environment triggering bacterial and host immune responses. The release of PG fragments from the bacterial cell can be caused by bacterial lysis events, resulting in the dispersion or active shedding and release from living bacteria. For example, *Neisseria* species release higher quantities of PG fragments into the extracellular environment during growth than other gram-negative bacteria (78–81).

When bacteria interact with a host, PG fragments function as pathogen-associated molecular patterns (PAMPs). These PAMPs are recognized by the host’s innate immune system and trigger defense mechanisms that cause an inflammatory response (for a review, see [82, 83]). The detection of PG fragments during infections is a vital sensing mechanism in most host systems, including animals (84), insects (85), and plants (86, 87). Muropeptides are recognized by host-produced receptors, such as the peptidoglycan recognition proteins (PGRP) and the NOD-like receptors. These PG receptors have distinct specificity toward specific muropeptide residues. For example, the receptor NOD1 recognizes the glutamate-*m*DAP moiety of the peptide side chain (57). In contrast, NOD2 recognizes muropeptides consisting of NAM with ʟ-ala, *iso*-ᴅ-glu of the peptide side chain, otherwise known as muramyl dipeptide (MDP) (82, 88, 89). Another unique PG receptor, a cytosolic hexokinase, detects NAG from PG to activate the NLRP3 inflammasome (90). In addition to detecting PG fragments, host-produced enzymes can also modify the structure of muropeptides. For example, recent work has shown that a host-produced *N*-acetylglucosamine kinase phosphorylates MDP to activate NOD2 signaling (91). By changing the structure of the PG muropeptides, these host-directed modifications could potentially alter or tune the response of the PGRP during the inflammatory response. Going forward, it will also be important to understand whether different hosts either sense, modify, or respond to muropeptides distinctly. For example, the mouse and human immune systems both exhibit a divergent response to another bacterial component, namely lipopolysaccharide (92).

To evade host defense mechanisms, bacterial species modify their muropeptides to interfere with PG receptor binding, reducing the host immune system response. For instance, O-acetylation or de-N-acetylation of the glycan chain (Fig. 2B, purple, blue) and the amidation of either the *iso*-ᴅ-glutamate or the *m*DAP (Fig. 2B, yellow, teal) on the peptide side chain limit recognition by the NOD receptors (89, 93–96). In addition, O-acetylation of the polysaccharide backbone (Fig. 2B, purple) limits binding and subsequent digestion by host-excreted lysozyme defensive enzymes (97). With a peptidoglycomic approach, all modifications to the PG that occur within the bacterial cell in response to the host can be identified. However, further method development for the purification of soluble muropeptides will be required to determine the breadth of released muropeptides that interact with the host. Currently, several techniques, including gel filtration, precipitation, or radiolabeling, have been used to identify soluble muropeptides released from bacteria into the media (80, 98–100). However, a metabolomic/peptidoglycomic methodology to identify the breadth of muropeptides released into the media has not been performed. To date, the soluble muropeptide purification methodology primarily focuses on isolating muropeptides from the growth media of monocultures. Transitioning to methodology using a host-bacterial environment will have many hurdles to overcome, including multi-bacterial species culture, breakdown of PG due to host immune responses, as well as identifying muropeptides among the multitude of other released metabolites or cellular debris. Understanding the entire conversation that occurs between bacteria and the host is crucial in understanding the role of the PG during these complex host-pathogen interactions.

Most bacterial species have PG as part of their cell wall. Therefore, when considering beneficial bacteria-host interactions, this landscape of host detection and pathogen immune evasion becomes more complicated. The recognition of friend vs foe is imperative in the intestinal microbiome, where the diversity of beneficial, commensal, and pathogenic bacterial species is highly complex, and dysbiosis has been implicated in many health-related inflammatory diseases (101–106).

Within the gut of flies (107, 108) and mice (109, 110), host-produced PGRPs influence the bacterial species maintained in the intestinal microbiome. For example, the loss of a PGRP in knockout mice increased the number of inflammation-promoting bacterial species within the intestinal microbiome (111). This reduced ability to control the abundance of pathogenic bacteria varies depending on the specific region of the intestinal tract (108). However, how PGRPs can influence the community composition of distinct bacteria and what role PG plays in that differentiation is unknown.

Recently, Harris-Jones et al. examined *Neisseria* species to determine whether PG influences beneficial and detrimental host-bacterial interactions (78). They determined that the level of PG fragment release did not distinguish between commensal and pathogenic *Neisseria* species (i.e., pathogenic species did not necessarily release increased levels of inflammatory PG). In addition, muropeptides derived from both species elicited similar NOD1 and NOD2 immune activation (78). They further showed that distinct *Neisseria* species released different types of muropeptides (78), suggesting that PG composition may be important. However, their methodology only broadly determined the PG composition. Therefore, a highly sensitive global peptidoglycomics approach (Fig. 3) would be beneficial in determining if there are specific muropeptide modifications that distinguish these *Neisseria* species. Including additional commensal and pathogenic *Neisseria* species would substantiate whether a particular modification of muropeptide influences host-bacterial interactions.

In another study, Onuma et al. found that the PG derived from two distinct commensal species (one gram-positive and one gram-negative) of the gut microbiota of *Drosophila melanogaster* elicited distinct host gene expression patterns (112). Interestingly, both have *m*DAP-containing PG structures, and neither was pathogenic to *D. melanogaster*. The gram-positive bacteria used in this study were *Lactiplantibacillus plantarum*, which contains a unique ᴅ-lactate moiety in position 5 (113) (Fig. 2B, pink). Whether the presence of this unique muropeptide modification contributed to changes in gene expression in *D. melanogaster* will need to be determined. A global peptidoglycomic approach could be advantageous in exploring these complex interactions between host and bacteria.

In addition to influencing PG receptor binding, peptidoglycan modifications may also impact host-bacterial interactions by affecting the uptake and systemic distribution of muropeptides. For example, could specific modifications attenuate the inflammatory response by limiting the infiltration of PG fragments into host cells? The uptake of bacterial-derived muropeptides across host membranes is mediated by proton-coupled oligopeptide transporters (POTs), which handle diverse substrates, including small peptides and drugs, such as β-lactams (114). Mammalian POT family members, PepT1 (SLC15A1), PepT2 (SLC15A2), Pht1 (SLC15A4), and Pht2 (SLC15A3), transport muropeptide fragments (115–118). For instance, pathogenic bacteria internalized via endocytosis release muropeptides that are transported by POTs from the endosome to the cytosol, where they interact with PG receptors and activate immune responses (119). Notably, PepT1 and PepT2 exhibit differences in peptide-uptake efficiencies influenced by the presence of certain di- or tripeptides (120, 121). However, it remains unclear whether muropeptide uptake efficiency varies with structural modifications to the peptide side chain or glycan backbone. Such variability may be an important consideration in understanding bacterial-host interactions, especially under conditions, such as stress or microbial dysbiosis, which can alter the permeability of the intestinal epithelial barrier and increase the PG fragment influx from the gut (122). Once absorbed, bacterial-derived muropeptides can translocate to distant sites within the host, including the blood, bone marrow (123), and even the brain, where they may influence development and behavior (124). Given that POT-mediated peptide transport is important in multiple organs, including the intestines (125), lungs, heart (126), and brain (127), it is essential to determine how PG modifications may affect the muropeptide uptake. Peptidoglycomics, with its ability to profile global PG composition and modifications, will play a pivotal role in dissecting these complex interactions. Going forward, the isolation and detection of released muropeptides could advance peptidoglycomic studies beyond just the cell-associated PG.

Much research remains to be done to understand bacterial-host interactions and the role PG plays in systemic benefits or detriments on host systems. Whether this interaction is beneficial or detrimental and whether this causality is due to receptor specificity, transport efficiency, or another yet unknown mechanism will remain to be seen. Regardless, having a clear picture of the entire breadth of the muropeptides interacting with a host via global peptidoglycomics will be a valuable tool in unraveling this complex association.

### Evolutionary insights into PG composition and physiological roles

An intriguing development in peptidoglycan research is the identification of ancestral peptidoglycan structures that are still present in some eukaryotic organelles. For instance, some plant species may retain remnants of peptidoglycan within their chloroplasts, reflecting their evolutionary origins as endosymbiotic cyanobacteria (128, 129). The muropeptide composition of chloroplast PG may even resemble that of gram-negative bacteria (130). This is consistent with findings in obligate intracellular pathogens, which often maintain reduced levels of PG (131). Applying peptidoglycomics to such atypical or vestigial PG systems could provide a sensitive and non-biased way to detect low-abundance or structurally divergent muropeptides, offering insight into the minimal requirements for PG biosynthesis and signaling. Mitochondria, another organelle with bacterial ancestry, can accumulate bacterial-derived muropeptides in both *Caenorhabditis elegans* (132) and human epithelial cells (133). The interaction of muropeptides with mitochondria stimulates ATP production and reduces oxidative stress, thereby benefiting the host cell. These findings raise intriguing questions: Do bacterial-derived muropeptides engage host mitochondria in a way that reflects ancestral bacterial physiology? Could the host’s ability to influence the selective growth of beneficial over pathogenic bacteria reflect an inter-bacterial competitive mechanism mediated by PG? Furthermore, what roles might PG modifications or inter-bacterial communication signals play in bacterial-host mitochondrial interactions? In these contexts, peptidoglycomics could profile bacterial PG composition and functionally characterize muropeptides interacting with host organelles, potentially shedding light on evolutionarily conserved recognition mechanisms.

## ADVANCING PEPTIDOGLYCOMICS: INNOVATIONS IN METHODOLOGY AND INTEGRATION

Future studies of the composition and physiological role of the PG will continue to benefit from technological improvements. Continued improvements to dedicated peptidoglycomics MS feature extraction and annotation software will significantly benefit the data analysis portion of the peptidoglycomic workflow. More efficient high-throughput methods for isolating PG sacculi are needed for peptidoglycomics analyses to be more widely applied. Several methodologies are emerging to fill this role, such as small culture volumes and a 96-well format (134, 135). However, these new techniques must demonstrate high-quality MS signals for similarly sensitive global peptidoglycomic analyses. The MS signal strength from the sacculi isolation in a 96-well format may be sufficient for the assessment of the highly abundant ‘core’ muropeptides in an undirected subset analysis. Whether the lower-abundant ‘adaptive’ muropeptides in global analyses are adequately covered using this sacculi isolation methodology remains to be determined.

Lastly, peptidoglycomics can be a powerful tool to help understand the cell wall’s stability and role in signaling. Integrating peptidoglycomics with other ‘omic’ technologies, such as proteomics, transcriptomics, or genomics, could be highly beneficial. For example, proteomics could validate a peptidoglycomic data set and indicate the proteins responsible for a specific muropeptide modification or the proteins affected by the presence of specific muropeptides. The combination of omics methodologies will help to clarify the production and modification of this complex biopolymer and shed light on its overall function in cell physiology.

## SUPPLEMENTAL MATERIAL

Table S1 jb.00359-25-s0001.docx Muropeptide modifications.

ASM does not own the copyrights to Supplemental Material that may be linked to, or accessed through, an article. The authors have granted ASM a non-exclusive, world-wide license to publish the Supplemental Material files. Please contact the corresponding author directly for reuse.
