# Supplementary material for: Compositional analysis of bacterial peptidoglycan: insights from peptidoglycomics into structure and function
Source: J Bacteriol. 2025 Oct 9;207(11):e00359-25. doi: 10.1128/jb.00359-25 (PMC12632261; doi:10.1128/jb.00359-25)
Supplement: Table S1 — Muropeptide modifications. [file jb.00359-25-s0001.docx]

**Table S1. Muropeptide modifications and the species that modify their peptidoglycan.** Alternate enzyme names are found in parentheses.

| **Position** | **Modification** | **Gram** | **Species** | **Enzymes responsible, if known (reference)** | **Acts on precursor / mature PG** | **predicted involvement in:** |
| --- | --- | --- | --- | --- | --- | --- |
| NAG or NAM | de-N-acetylation | pos. | *Bacillus anthracis* | BA1961, BA3679 [mod. of NAG], BA1977, BA2944, BA5436 (1, 2) | mature | capsule formation (3) |
|  |  | pos. | *Bacillus subtilis* | PdaA [mod. of NAM], PdaB, PdaC [mod. of NAM and NAG] (4–7) | mature |  |
|  |  | pos. | *Clostridioides difficile* | PgdA (CD630_15220), PgdB (CD630_32570), PdaV (CD630_15560), PdaA1 (CD630_14300) PdaA2 (CD630_27190) (8–10) | mature | lysozyme resistance (8, 10) sporulation/heat resistance (9) |
|  |  | pos. | *Listeria monocytogenes* | PgdA [mod. of NAG] (11, 12) | mature | lysozyme resistance (11) |
|  |  | pos. | *Mycobacterium tuberculosis* | Rv1096 (13) | mature |  |
|  |  | pos. | *Streptococcus pneumoniae* | PgdA [mod. of NAG](14) | mature |  |
|  |  | neg. | *Agrobacterium tumefaciens* | MdaA [mod. of 1,6-anhNAM] (15) | mature |  |
|  |  | neg. | *Bdellovibrio bacteriovorus* | Bd0468, Bd3279 (16) | mature | predation (16) |
|  |  | neg. | *Helicobacter pylori* | PgdA (17) | mature |  |
|  |  | neg. | *Legionella pneumophila* | PgdA (Lpg1993), Lpg0633 [mod. of NAG] (18) | mature | type IV secretion (18) |
|  | de-O-acetylation | neg. | *Campylobacter jejuni* | Ape (19) | mature |  |
|  |  | neg. | *Neisseria gonorrheae* | ApeI (20) | mature |  |
|  |  | neg. | *Neisseria meningitidis* | ApeI (21) | mature |  |
| NAG or NAM | O-acetylation | pos. | *Staphylococcus aureus* | OatA [mod. of NAM] (22) | mature | lysozyme resistance |
|  |  | pos. | *Streptococcus pneumoniae* | OatA, Adr [mod. of NAM] (23, 24) | mature | lysozyme resistance |
| NAG or NAM | O-acetylation | pos. | *Latilactobacillus plantarum* | OatA, OatB [mod. of NAG] (25) | mature | lysozyme resistance |
|  |  | pos. | *Listeria monocytogenes* | OatA (26) | mature | lysozyme resistance |
|  |  | neg. | *Neisseria gonorrhoeae* | PatA, PatB (27, 28) | mature | lysozyme resistance |
|  |  | neg. | *Neisseria meningitidis* | PatA, PatB (21) | mature | lysozyme resistance |
|  |  | neg. | *Helicobacter pylori* | PatA (29) | mature | lysozyme resistance |
|  |  | neg. | *Campylobacter jejuni* | PatA and PatB (19) | mature | lysozyme resistance |
| NAM | 1,6-anhydro | pos. | *Bacillus subtilis* | SleB – [digests δ-lactam NAM], SpoIID, CwlQ (30–32) | mature | sporulation (31) |
|  |  | pos. | *Caulobacter crescentus* | SdpA, SdpB,SdpC, PleA (33, 34) | mature | division (33), flagella assembly (34) |
|  |  | pos. | *Clostridioides difficile* | SleC, SpoIID (35, 36) | mature | sporulation (35, 36) |
|  |  | pos. | *Mycobacterium abscessus* | maMltG (37) | mature |  |
|  |  | pos. | *Staphylococcus aureus* | LsaA, SceD (38) | mature | biofilm / antibiotic resistance (38) |
|  |  | neg. | *Acinetobacter baumannii* | MltA, MltB, MltD, MltF, MltG, Slt, SltB, Tae17 (39–42) | mature | T6SS effector (39) |
|  |  | neg. | *Agrobacterium tumefaciens* | MltA (atu0009), MltB1 (atu0092), MltB2 (atu2122), MltB3 (atu3779), Slt1 (atu1022), Slt2 (adu2112), Slt3, (atu2117), Slt4 (atu3093) (43) | mature | AmpC expression (43) |
|  |  | neg. | *Bdellovibrio bacteriovorus* | Bd3285 (44) | mature | predation (44) |
|  |  | neg. | *Borrelia burgdorferi* | MltS (BB0259) (45) | mature | host immune evasion |
| NAM | 1,6-anhydro | neg. | *Escherichia coli* | MltA, MltB, MltC, MltD, MltE, MltF, MltG, Slt, RlpA (review (46, 47)) | mature | PG recycling |
|  |  | neg. | *Francisella tularensis* | Slt, MltA (48) | mature |  |
|  |  | neg. | *Myxococcus xanthus* | AgmT (49) | mature | motility (49) |
|  |  | neg. | *Neisseria gonorrhoeae* | LtgA, LtgB, LtgD, MltA (GNA33), MltG, ltgX , AltA (50–55) | mature | PG release (54) |
|  |  | neg. | *Pseudomonas aeruginosa* | MltA, MltB, MltD, MltF, MltF2, MltG, Slt, SltB2, SltH(SltB), RlpA (review: (47)) | mature | PG recycling |
|  |  | neg. | *Pseudomonas syringae* | HrpH (56) | mature | T3SS - plant immune interaction (56) |
|  |  | neg. | *Rhodobacter sphaeroides* | SltF (57) | mature | flagella assembly (57) |
|  |  | neg. | *Vibrio cholerae* | MltA, MltB, MltC, MltD, MltF, MltG, Slt70, RlpA (58) | mature |  |
|  |  | neg. | *Xanthomonas citri* | 16 putative LTs including XAC4296 a fused lytic transglycosylase and epimerase (59, 60) | mature |  |
|  |  |  | bacteriophage T7 | Gp16 (61) | mature | virulence (61) |
|  |  |  | bacteriophage BFK20 | Slt (62) | mature | virulence (62) |
|  |  |  | *Physcomitrella patens (moss)* | MltB (63) | mature | chloroplast division |
| NAM | N-glycolyl | pos. | *Microbacterium aquilitoris*  */ Microbacterium gawkjiense* (64) |  |  |  |
|  |  | pos. | *Motilibacter rhizosphaerae* (65) |  |  |  |
|  |  | pos. | *Mycobacterium leprae* | non-functional NamH (66) |  |  |
|  |  | pos. | *Mycobacterium tuberculosis* | NamH (Rv3818) (67) | precursor | immunogenicity (NOD2) (67) |
|  |  |  |  |  |  |  |
| NAM | N-glycolyl | pos. | *Mycobacterium smegmatis* | NamH (MSMEG_6410) (68, 69) | precursor | β-lactam resistance  (68, 69) |
|  |  | pos. | *Nocardia asteroides* (70) |  |  |  |
| NAM | δ-lactam | pos. | *Bacillus subtilis* | CwlD. PdaA (71) | mature |  |
|  |  | pos. | *Clostridioides difficile* | CwlD, PdaA1 (CD630_14300), PdaA2 (CD630_27190) (9) | mature |  |
| first amino acid | glycine | pos. | *Staphylococcus aureus* | MurC (72) | precursor | species specific |
|  |  | pos. | *Mycobacterium tuberculosis* and *Mycobacterium leprae* | Rv2152c, MLCB268.01c (73) | precursor | species specific |
|  |  | neg. | *Chlamydia trachomatis* | MurC (74) | precursor | species specific |
|  |  | neg. | *Escherichia coli* | MurC (75) | precursor | species specific |
| first amino acid | L-serine | pos. | *Staphylococcus aureus* | MurC (72) | precursor | species specific |
|  |  | neg. | *Chlamydia trachomatis* | MurC (74) | precursor | species specific |
|  |  | neg. | *Escherichia coli* | MurC (75) | precursor | species specific |
| second amino acid | D-glutamine | pos. | *Lactococcus lactis* | GatD, MurT (76) | precursor | acid resistance (76) |
|  |  | pos. | *Geobacillus stearothermophilus* (77) |  | precursor |  |
|  |  | pos. | *Mycobacterium tuberculosis* | MurT (Rv3712), GatD (Rv3713) (78) | precursor | immune evasion (79) |
|  |  | pos. | *Mycobacterium smegmatis* | GatD (MSMEG_6277), MurT (MSMEG_6276) (69) | precursor | crosslinking (80) |
|  |  | pos. | *Staphylococcus aureus* | GatD (SA1707), MurT (SA1708) (81) | precursor | species specific |
|  |  | pos. | *Streptococcus pneumoniae* | GatD, MurT (82) | precursor | crosslinking (83) |
| second amino acid | hydroxy-glutamate | pos. | *Agreia bicolorata* (84) |  |  | species specific |
|  |  | pos. | *Cnuibacter physcomitrellae* (85) |  |  | species specific |
| second amino acid | hydroxy-glutamate | pos. | *Ilumatobacter fluminis* (86) |  |  | species specific |
|  |  | pos. | *Microbacterium arthrosphaerae* (87) |  |  | species specific |
|  |  | pos. | *Microbacterium insulae* (88) |  |  |  |
|  |  | pos. | *Microbacterium lacticum* (89) |  | mature (89) | species specific |
|  |  | pos. | *Microbacterium lemovicicum* (90) |  |  | species specific |
|  |  | pos. | *Microbacterium telephonicum* (91) |  | mature (91) | species specific |
|  |  | pos. | *Microbacterium yannicii* (92) |  |  | species specific |
| second amino acid | appended amino acids / polyamides |  |  |  |  |  |
|  | D-alaninamide | pos. | *Arthrobacter sp.NCIB9423* (93) |  |  | species specific |
|  | glycinamide | pos. | *Arthrobacter atrocyaneus* (94) |  |  | species specific |
|  | glycinamide | pos. | coryneform isolates, CNRZ 925 and CNRZ 926 (95) |  |  | species specific |
|  | glycine/serine | pos. | *Micrococcus lysodeikticus* (96, 97) |  |  | species specific |
|  | glycine | pos. | *Eubacterium nodatum* (98) |  |  | species specific |
|  | spermidine / cadaverine | neg. | *Anaerovibrio lipolytica* (99) |  |  | species specific |
|  | agmatine | neg. | *Selenomonas flueggei* and *S. sputigena* (100) |  |  | species specific |
|  | cadaverine | neg. | *Selenomonas ruminantium* | lipid intermediate diamine transferase (101) | precursor | anchorage of PG to outer membrane (102) |
| second amino acid | putrescine / cadaverine | neg. | Veillonella alcalescens, *V. parvula* (103) |  |  | species specific |
|  | N-acetyl putrescine |  | *Glaucocystis nostochinearum, Cyanoptyche gloeocystis, Cyanophora paradoxa* (algae / glaucophyte)  (104, 105) |  |  | species specific |
| third amino acid | *m*DAP | pos. | *Bacillus subtilis* | MurE (106) | precursor | species specific |
|  | *m*DAP | pos. | *Mycobacterium thermoresistibile* | MthMurE (107) | precursor | species specific |
|  | *m*DAP | pos. | *Mycobacterium tuberculosis* | MtbMurE (Rv2158c) (108) | precursor | species specific |
|  | *m*DAP | neg. | *Acinetobacter baumannii* | MurE (109) | precursor | species specific |
|  | *m*DAP | neg. | *Bordetella pertussis* | MurE–MurF chimera (110) | precursor | species specific |
|  | *m*DAP | neg. | *Chlamydia trachomatis* | MurE (111) | precursor | species specific |
|  | *m*DAP | neg. | *Escherichia coli* | MurE (112, 113) | precursor | species specific |
|  | *m*DAP | neg. | *Haemophilus influenzae* | MurE (114) | precursor | eDNA uptake (114) |
|  | *m*DAP | neg. | *Fusobacterium sulci,*  *F. ulcerans, F. varium* (115) |  | precursor | species specific |
|  | *m*DAP | neg. | *Pseudomonas aeruginosa* | MurE (116) | precursor | species specific |
|  | *m*DAP | neg. | *Salmonella enterica serovar Typhi* | StMurE (117) | precursor | species specific |
|  | *m*DAP | neg. | *Verrucomicrobium spinosum* | MurE (118) | precursor | species specific |
|  | unknown |  | *Larix gmelinii* (plant - larch) | MurE (119) | precursor | chloroplast development |
|  | unknown |  | *Arabidopsis thaliana* (plant) | MurE (120) | precursor | chloroplast development |
| third amino acid | *m*DAP (121) |  | *Physcomitrella patens* (moss) | MurE (122) | precursor | chloroplast division |
|  | L-lysine | pos. | *Corynebacterium glutamicum* | MurE (123) | precursor | species specific |
|  | L-lysine | pos. | *Enterococcus faecium* (124) |  | precursor | species specific |
|  | L-lysine | pos. | *Staphylococcus aureus* | MurE (72, 125) | precursor | species specific |
|  | L-lysine | pos. | *Streptococcus pneumoniae* | MurE (126) | precursor | species specific |
|  | alanine / *m*DAP | pos. | *Erysipelothrix rhusiopathiae* | MurE (127) | precursor | species specific |
|  | D-lysine | neg. | *Thermotoga maritima* | MurE (TM1597) (128, 129) | precursor | species specific |
|  | *m*DAP / lysine | neg. | *Microcystis aeruginosa* | MurE (130) | precursor | species specific |
|  | *meso*-lanthionine | pos. | Romboutsia lituseburensis (131) |  | precursor | species specific |
|  | *meso*-lanthionine | neg. | *Fusobacterium* sp. (115, 132, 133) |  | precursor(134) | species specific |
|  |  |  | ***Spirochetes:*** |  |  |  |
|  | L-ornithine | neg. | *Spirochaeta stenostrepta, S. auranti* (135) |  | precursor | species specific |
|  | L-ornithine | neg. | *Borrelia burgdorferi* (136) |  | precursor | species specific |
|  | L-ornithine | neg. | *Treponema pallidum* (137) |  | precursor | species specific |
|  | L-ornithine | neg. | *Salinispira pacifica* (138) |  | precursor | species specific |
|  |  |  | **Actinobacteria:** |  |  |  |
|  | L-ornithine / lysine | pos. | *Actinomyces* sp. (139, 140) |  | precursor | species specific |
|  | L-ornithine | pos. | *Bifidobacterium globosum* (141) |  | precursor | species specific |
|  | L-ornithine | pos. | *Cellulomonas* sp. (142–144) |  | precursor | species specific |
|  | L-ornithine | pos. | *Microbacterium* sp. (145–147) |  | precursor | species specific |
| third amino acid | L-ornithine | pos. | *Ornithinimicrobium* sp. (148, 149) |  | precursor | species specific |
|  |  |  | **Firmicutes:** |  |  |  |
|  | L-ornithine | pos. | *Eubacterium* sp. (98, 150) |  | precursor | species specific |
|  | L-ornithine | pos. | *Bacillus ciccensis* (151) |  | precursor | species specific |
|  | L-ornithine / lysine | pos. | *Oceanobacillus piezotolerans* (152) |  | precursor | species specific |
|  | L-ornithine | pos. | *Ornithinibacillus caprae* (153) |  | precursor | species specific |
|  | L-ornithine | neg. | *Alkalibacillus aidingensis* (154) |  | precursor | species specific |
|  |  |  | **Deinococcota:** |  |  |  |
|  | L-ornithine | neg. | *Deinococcus* sp. (155–157) |  | precursor | species specific |
|  | L-ornithine | neg. | *Thermus thermophilus* (158) |  | precursor | species specific |
|  |  |  | **Chloroflexota:** |  |  |  |
|  | L-ornithine | pos. | *Dictyobacter halimunensis* (159) |  | precursor | species specific |
|  | L-ornithine | neg. | *Kallotenue papyrolyticum* (160) |  | precursor | species specific |
|  | L-ornithine | pos. | *Ktedonosporobacter rubrisoli* (161) |  | precursor | species specific |
|  | L-ornithine | neg. | *Oscillochloris trichoides* (162) |  | precursor | species specific |
| third amino acid | amidated *m*DAP | pos. | *Bacillus subtilis* | AsnB (163) | precursor |  |
|  |  | pos. | *Clostridioides difficile* | AsnB (164) | precursor |  |
|  |  | pos. | *Corynebacterium glutamicum* | LtsA (Cg2410) (165) |  | lysozyme resistance (165) |
|  |  | pos. | *Latilactobacillus plantarum* | AsnB1(166) | precursor |  |
| third amino acid | amidated *m*DAP | pos. | *Listeria monocytogenes* | AsnB (167) | precursor | host invasion / motility (167) |
|  |  | pos. | *Mycobacterium tuberculosis* | AsnB (168) | precursor |  |
| fourth amino acid | glycine, non-canonical D-amino acid | pos. | *Corynebacterium callunae* and *Bacillus subtilis* |  |  | PG synthesis / dec. crosslink (169, 170) |
|  |  | pos. | *Mycobacterium smegmatis* | *ldtA(MSMEG_3528),ldtB(MSMEG_4745), ldtE(MSMEG_0233)* (171) | mature |  |
|  |  | pos. | *Salmonella enterica* serovar Typhimurium | Tlde1a (172, 173) | mature | bacterial competition (172, 173) |
|  |  | neg. | *Acinetobacter baumannii* |  |  | bacterial competition (174) |
|  |  | neg. | *Escherichia coli* | LdtA(ErfK), LdtB(YbiS), LdtC(YcfS), LdtD(YcbB) LdtE(YnhG) (175, 176) | mature |  |
|  |  | neg. | *Vibrio cholerae* | LdtA (Vc1268), LdtB (Vca0058) (176, 177) | mature | environmental adaptation, PG signalling (170, 178) |
| fourth amino acid | alaninol | pos. | *Salmonella enterica* serovar *Typhimurium* (179) |  |  | host immune evasion (179) |
|  |  | pos. | *Mycobacterium smegmatis* (180) |  |  |  |
| fourth amino acid | methylated D-alanine | pos. | *Mycobacterium smegmatis* (180) |  |  |  |
|  |  | neg. | *Pseudomonas aeruginosa* (181) |  |  |  |
|  |  |  |  |  |  |  |
| fifth amino acid | glycine, non-canonical D-amino acid | pos. | *Bacillus subtilis* | DD-transpeptidase, Pbp1 (176, 182) | mature |  |
|  |  | pos. | *Staphylococcus aureus* | PBP4 (183) | mature | decreased crosslink (169) |
|  |  | neg. | *Escherichia coli* | MrcA (Pbp1A), MrcB (Pbp1B) (170, 176, 184) | mature |  |
|  |  | neg. | *Vibrio cholerae* | Ddl/MurF (177) | precursor |  |
| fifth amino acid | D-lactate | pos. | *Enterococcus faecalis* | VanA, VanB, VanD (185, 186) | precursor | Antimicrobial resistance (185, 186) |
|  |  | pos. | *Enterococcus faecium* | VanA, VanD, VanM (186–188) | precursor | Antimicrobial resistance (186–188) |
|  |  | pos. | *Latilactobacillus plantarum* | Ddl/MurF (189) | precursor | immune response (189) |
|  |  | pos. | *Staphylococcus aureus* | VanA, VanB (190–192) | precursor | Antimicrobial resistance |
|  |  | pos. | *Streptomyces coelicolor* | VanA (SCO3595) (193) | precursor | Antimicrobial resistance |
|  |  | pos. | *Streptomyces toyocaensis* | *DdlM, VanA* (194, 195) | precursor | Antimicrobial resistance |
|  |  | pos. | *Amycolaptosis orientalis* | *DdlN, VanA* (194, 195) | precursor | Antimicrobial resistance |
| fifth amino acid | D-serine | pos. | *Clostridioides difficile* | VanG_Cd_ (196) | precursor | Antimicrobial resistance |
|  |  | pos. | *Enterococcus gallinarum* | VanC, VanC-1 (197, 198) |  | Antimicrobial resistance |
|  |  | pos. | *Enterococcus casseliflavus* | VanC-2, VanC-3, VanC-4 (199–201) |  | Antimicrobial resistance |
|  |  | pos. | *Enterococcus faecalis* | VanC1, VanE, VanG, VanL (202–207) |  | Antimicrobial resistance |
|  |  | pos. | *Enterococcus faecium* | VanN (208) |  | Antimicrobial resistance |
| fifth amino acid | D-serine | pos. | *Enterococcus flavescens* | VanC-3 (201) |  | Antimicrobial resistance |
|  |  | pos. | *Lactococcus lactis* | MurF involved (209) | precursor | acid resistance, nisin yield (209) |
| crosslinks | 3-4 | pos. | *Mycobacterium tuberculosis* | PonA2 (Pbp1a/rv3682), PonA1 (Pbp1b/rv0050), PbpA (Pbp2/rv0016c) (210) | mature | PG synthesis |
|  |  | pos. | *Staphylococcus aureus* | Pbp1, Pbp2, Pbp3, Pbp4 (211) | mature | PG synthesis |
|  |  | pos. | *Streptococcus pneumoniae* | Pbp1a, Pbp1b, Pbp2a, Pbp2b, Pbp2x (212) | mature | PG synthesis |
|  |  | neg. | *Escherichia coli* | MrcA (Pbp1A), MrcB (Pbp1B), PbpC (Pbp1C), MrdA (Pbp2), FtsI (Pbp3) (213) | mature | PG synthesis |
|  |  | neg. | *Pseudomonas aeruginosa* | PonA (Pbp1A), MrcB (Pbp1B), PbpA (Pbp2), FtsI (Pbp3), Pbp3a (213) | mature | PG synthesis |
|  |  |  | *Physcomitrium patens (moss)* | PpPBP (122) | mature | PG synthesis / chloroplast div. |
|  |  |  | Helpful reviews:  (214–217) |  |  |  |
| crosslinks | 3-3 | pos. | *Clostridioides difficile* | Ldt_Cd1_, Ldt_Cd2_, Ldt4, Ldt5 (218, 219) | mature |  |
|  |  | pos. | *Enterococcus faecium* | Ldt_Fm_ (220) | mature |  |
|  |  | pos. | *Mycobacterium tuberculosis* | Ldt_Mt1_ (Rv0116c), Ldt_Mt2_ (Rv2518c),  Ldt_Mt3_ (Rv1433) (221–223) | mature |  |
|  |  | pos. | *Mycobacterium smegmatis* | LdtE (MSMEG_0233) (171) | mature |  |
|  |  | pos. | *Salmonella enterica* | LdtD (YcbB), LdtE (YnhG) (179) | mature |  |
|  |  | neg. | *Acinetobacter baumannii* | Ldt_Ab_(LdtJ) (224) | mature |  |
|  |  | neg. | *Agrobacterium tumefaciens* | Atu0048, Atu0669, Atu0844, Atu0845, Atu2336, Atu3331, Atu5196 (225) | mature |  |
|  |  | neg. | *Escherichia coli* | LdtD (YcbB), LdtE (YnhG) (175) | mature |  |
|  |  | neg. | *Legionella pneumophila* | Lpg1386 (226) | mature |  |
|  |  | neg. | *Pseudomonas aeruginosa* | Ldt_pae1_(PA14_54810) (227) | mature |  |
| crosslinks | 1-3 | neg. | *Gluconobacter oxydans* | Ldt_Go_ (228) | mature |  |
| crosslinks | amino acid bridge |  |  |  |  |  |
|  | Ala-Ala | pos. | *Enterococcus faecalis* | BppA1, BppA2 (229) |  |  |
|  | D-Asp | pos. | *Enterococcus faecium* | Asl_fm_ (230) | precursor |  |
|  | D-Asp | pos. | *Lactococcus lactis* | AslA (YxbA) (231) |  |  |
|  | Gly-Gly-Gly-Gly-Gly | pos. | *Staphylococcus aureus* | FemA, FemB, FmhB (FemX) (232, 233) | precursor (234) |  |
|  | Gly-Gly-Ser-Gly-Ser | pos. | *Staphylococcus aureus* | FmhA, FmhC (235) |  | bacteriocin resistance (235) |
|  | Ala | pos. | *Streptococcus oralis* | MurM (236) |  |  |
|  | Ala-Ser / Ala-Ala | pos. | *Streptococcus pneumoniae* | MurM, MurN (237) | precursor | penicillin resistance (237) |
|  | Ala-Ala-Ala | pos. | *Streptococcus equi* | *Zif* (238) |  | bacterial competition (238) |
|  | Gly | pos. | Streptomyces coelicolor | VanK (SCO3593) (193, 239) |  | Glycopeptide resistance (193) |
|  | Ala-Ser / Ala-Ser-Ala | pos. | *Weissella viridescens* | FemX_Wv_ (240) |  |  |

REFERENCES – Table S1

1. Balomenou S, Fouet A, Tzanodaskalaki M, Couture-Tosi E, Bouriotis V, Boneca IG. 2013. Distinct functions of polysaccharide deacetylases in cell shape, neutral polysaccharide synthesis and virulence of *Bacillus anthracis*. Mol Microbiol 87:867–883.

2. Andreou A, Giastas P, Christoforides E, Eliopoulos EE. 2018. Structural and evolutionary insights within the polysaccharide deacetylase gene family of *Bacillus anthracis* and *Bacillus cereus*. Genes (Basel) 9:386.

3. Candela T, Balomenou S, Aucher W, Bouriotis V, Simore JP, Fouet A, Boneca IG. 2014. *N*-acetylglucosamine deacetylases modulate the anchoring of the gamma-glutamyl capsule to the cell wall of *Bacillus anthracis*. Microb Drug Resist 20:222–230.

4. Fukushima T, Kitajima T, Sekiguchi J. 2005. A polysaccharide deacetylase homologue, PdaA, in *Bacillus subtilis* acts as an *N*-acetylmuramic acid deacetylase in vitro. J Bacteriol 187:1287–1292.

5. Fukushima T, Tanabe T, Yamamoto H, Hosoya S, Sato T, Yoshikawa H, Sekiguchi J. 2004. Characterization of a polysaccharide deacetylase gene homologue (*pdaB*) on sporulation of *Bacillus subtilis*. J Biochem 136:283–291.

6. Kobayashi K, Putu Sudiarta I, Kodama T, Fukushima T, Ara K, Ozaki K, Sekiguchi J. 2012. Identification and characterization of a novel polysaccharide deacetylase C (PdaC) from *Bacillus subtilis*. J Biol Chem 287:9765–9776.

7. Fukushima T, Yamamoto H, Atrih A, Foster SJ, Sekiguchi J. 2002. A polysaccharide deacetylase gene (*pdaA*) is required for germination and for production of muramic δ-lactam residues in the spore cortex of *Bacillus subtilis*. J Bacteriol 184:6007–6015.

8. Kaus GM, Snyder LF, Müh U, Flores MJ, Popham DL, Ellermeier CD. 2020. Lysozyme resistance in *Clostridioides difficile* is dependent on two peptidoglycan deacetylases. J Bacteriol 202:1–15.

9. Coullon H, Rifflet A, Wheeler R, Janoir C, Boneca IG, Candela T. 2018. *N*-Deacetylases required for muramic-δ-lactam production are involved in *Clostridium difficile* sporulation, germination, and heat resistance. J Biol Chem 293:18040–18054.

10. Coullon H, Rifflet A, Wheeler R, Janoir C, Boneca IG, Candela T. 2020. Peptidoglycan analysis reveals that synergistic deacetylase activity in vegetative *Clostridium difficile* impacts the host response. J Biol Chem 295:16785–16796.

11. Boneca IG, Dussurget O, Cabanes D, Nahori MA, Sousa S, Lecuit M, Psylinakis E, Bouriotis V, Hugot JP, Giovannini M, Coyle A, Bertin J, Namane A, Rousselle JC, Cayet N, Prévost MC, Balloy V, Chignard M, Philpott DJ, Cossart P, Girardin SE. 2007. A critical role for peptidoglycan N-deacetylation in *Listeria* evasion from the host innate immune system. Proc Natl Acad Sci U S A 104:997–1002.

12. Popowska M, Kusio M, Szymañska P, Markiewicz Z. 2009. Inactivation of the wall-associated de-*N*-acetylase (PgdA) of *Listeria monocytogenes* results in greater susceptibility of the cells to induced autolysis. J Microbiol Biotechnol 19:932–945.

13. Yang S, Zhang F, Kang J, Zhang W, Deng G, Xin Y, Ma Y. 2014. *Mycobacterium tuberculosis* Rv1096 protein: Gene cloning, protein expression, and peptidoglycan deacetylase activity. BMC Microbiol 14:1–9.

14. Vollmer W, Tomasz A. 2000. The pgdA gene encodes for a peptidoglycan N-acetylglucosamine deacetylase in *Streptococcus pneumoniae*. J Biol Chem 275:20496–20501.

15. Gilmore MC, Yadav AK, Espaillat A, Gust AA, Williams MA, Brown PJB, Cava F. 2024. A peptidoglycan N-deacetylase specific for anhydroMurNAc chain termini in *Agrobacterium tumefaciens*. J Biol Chem 300:105611.

16. Lambert C, Lerner TR, Bui NK, Somers H, Aizawa SI, Liddell S, Clark A, Vollmer W, Lovering AL, Sockett RE. 2016. Interrupting peptidoglycan deacetylation during *Bdellovibrio* predator-prey interaction prevents ultimate destruction of prey wall, liberating bacterial-ghosts. Sci Rep 6:1–19.

17. Wang G, Olczak A, Forsberg LS, Maier RJ. 2009. Oxidative stress-induced peptidoglycan deacetylase in *Helicobacter pylori*. J Biol Chem 284:6790–6800.

18. Boamah D, Gilmore MC, Bourget S, Ghosh A, Hossain MJ, Vogel JP, Cava F, O’Connor TJ. 2023. Peptidoglycan deacetylation controls type IV secretion and the intracellular survival of the bacterial pathogen *Legionella pneumophila*. Proc Natl Acad Sci 120:e2119658120.

19. Ha R, Frirdich E, Sychantha D, Biboy J, Taveirne ME, Johnson JG, Di Rita VJ, Vollmer W, Clarke AJ, Gaynor EC. 2016. Accumulation of peptidoglycan O-acetylation leads to altered cell wall biochemistry and negatively impacts pathogenesis factors of *Campylobacter jejuni*. J Biol Chem 291:22686–22702.

20. Weadge JT, Clarke AJ. 2006. Identification and characterization of O-acetylpeptidoglycan esterase: A novel enzyme discovered in *Neisseria gonorrhoeae*. Biochemistry 45:839–851.

21. Veyrier FJ, Williams AH, Mesnage S, Schmitt C, Taha MK, Boneca IG. 2013. De-O-acetylation of peptidoglycan regulates glycan chain extension and affects in vivo survival of *Neisseria meningitidis*. Mol Microbiol 87:1100–1112.

22. Bera A, Herbert S, Jakob A, Vollmer W, Götz F. 2005. Why are pathogenic staphylococci so lysozyme resistant? The peptidoglycan O-acetyltransferase OatA is the major determinant for lysozyme resistance of *Staphylococcus aureus*. Mol Microbiol 55:778–787.

23. Crisóstomo MI, Vollmer W, Kharat AS, Inhülsen S, Gehre F, Buckenmaier S, Tomasz A. 2006. Attenuation of penicillin resistance in a peptidoglycan O-acetyl transferase mutant of *Streptococcus pneumoniae*. Mol Microbiol 61:1497–1509.

24. Sychantha D, Clarke AJ. 2018. Peptidoglycan modification by the catalytic domain of *Streptococcus pneumoniae* OatA follows a ping-pong Bi-Bi mechanism of action. Biochemistry 57:2394–2401.

25. Bernard E, Rolain T, Courtin P, Guillot A, Langella P, Hols P, Chapot-Chartier MP. 2011. Characterization of O-acetylation of *N*-acetylglucosamine: A novel structural variation of bacterial peptidoglycan. J Biol Chem 286:23950–23958.

26. Aubry C, Goulard C, Nahori MA, Cayet N, Decalf J, Sachse M, Boneca IG, Cossart P, Dussurget O. 2011. OatA, a peptidoglycan O-acetyltransferase involved in *Listeria monocytogenes* immune escape, is critical for virulence. J Infect Dis 204:731–740.

27. Weadge JT, Pfeffer JM, Clarke AJ. 2005. Identification of a new family of enzymes with potential O-acetylpeptidoglycan esterase activity in both Gram-positive and Gram-negative bacteria. BMC Microbiol 5:1–15.

28. Moynihan PJ, Clarke AJ. 2010. O-acetylation of peptidoglycan in gram-negative bacteria: Identification and characterization of peptidoglycan O-acetyltransferase in *Neisseria gonorrhoeae*. J Biol Chem 285:13264–13273.

29. Wang G, Lo LF, Forsberg LS, Maier RJ. 2012. *Helicobacter pylori* peptidoglycan modifications confer lysozyme resistance and contribute to survival in the host. MBio 3:1–9.

30. Heffron JD, Sherry N, Popham DL. 2011. In vitro studies of peptidoglycan binding and hydrolysis by the *Bacillus anthracis* germination-specific lytic enzyme SleB. J Bacteriol 193:125–131.

31. Marathe A, Zarazúa-Osorio B, Srivastava P, Fujita M. 2023. The master regulator for entry into sporulation in *Bacillus subtilis* becomes a mother cell-specific transcription factor for forespore engulfment. Mol Microbiol 120:439–461.

32. Sudiarta IP, Fukushima T, Sekiguchi J. 2010. *Bacillus subtilis* CwlQ (previous YjbJ) is a bifunctional enzyme exhibiting muramidase and soluble-lytic transglycosylase activities. Biochem Biophys Res Commun 398:606–612.

33. Zielińska A, Billini M, Möll A, Kremer K, Briegel A, Izquierdo Martinez A, Jensen GJ, Thanbichler M. 2017. LytM factors affect the recruitment of autolysins to the cell division site in *Caulobacter crescentus*. Mol Microbiol 106:419–438.

34. Viollier PH, Shapiro L. 2003. A lytic transglycosylase homologue, PleA, is required for the assembly of pili and the flagellum at the *Caulobacter crescentus* cell pole. Mol Microbiol 49:331–345.

35. Kim C, Molina R, Lee M, Garay-alvarez A, Yang J, Qian Y, Birhanu BT, Hesek D, Hermoso JA, Chang M, Mobashery S. 2025. Reactions of SleC, its structure and inhibition in mitigation of spore germination in *Clostridioides difficile*. J Am Chem Soc 147:5060–5070.

36. Nocadello S, Minasov G, Shuvalova LS, Dubrovska I, Sabini E, Anderson WF. 2016. Crystal structures of the SpoIID lytic transglycosylases essential for bacterial sporulation. J Biol Chem 291:14915–14926.

37. Lee GH, Kim S, Kim DY, Han JH, Lee SY, Lee JH, Lee CS, Park HH. 2024. Structure of MltG from *Mycobacterium abscessus* reveals structural plasticity between composed domains. IUCrJ 11:903–909.

38. Lopes A-A, Yoshii Y, Yamada S, Nagakura M, Kinjo Y, Mizunoe Y, Okudaa K. 2019. Roles of lytic transglycosylases in biofilm formation and β-Lactam resistance in methicillin-resistant *Staphylococcus aureus*. Antimicrob Agents Chemother 63:e01277-19.

39. Bezkorovayna V, Hayes BK, Gillett FN, Wright A, Roper DI, Harper M, McGowan S, Boyce JD. 2025. Delivery determinants of an *Acinetobacter baumannii* type VI secretion system bifunctional peptidoglycan hydrolase. MBio 16:e02627-24.

40. Jang H, Do H, Kim CM, Kim GE, Lee JH, Parka HH. 2021. Molecular basis of dimerization of lytic transglycosylase revealed by the crystal structure of MltA from *Acinetobacter baumannii*. IUCrJ 8:921–930.

41. Jang H, Kim CM, Ha HJ, Hong E, Park HH. 2024. Interdomain flexibility and putative active site was revealed by crystal structure of MltG from *Acinetobacter baumannii*. Biochem Biophys Res Commun 727:150318.

42. Critchlow JM, Barraza JP, Munneke MJ, Krystofiak E, Green ER, Skaar EP. 2025. The interplay between *Acinetobacter baumannii* ZigA and SltB promotes zinc homeostasis and cell envelope integrity. Infect Immunityt 93:e0042224.

43. Figueroa-Cuilan WM, Howell M, Richards C, Randich A, Yadav AK, Cava F, Brown PJB. 2022. Induction of AmpC-mediated β-lactam resistance requires a single lytic transglycosylase in *Agrobacterium tumefaciens*. Appl Environ Microbiol 88:e00333-22.

44. Banks EJ, Lambert C, Mason SS, Tyson J, Radford PM, McLaughlin C, Lovering AL, Sockett RE. 2023. An MltA-like lytic transglycosylase secreted by *Bdellovibrio bacteriovorus* cleaves the prey septum during predatory invasion. J Bacteriol 205:e0047522.

45. McCausland JW, Kloos ZA, Irnov I, Sonnert ND, Zhou J, Putnick R, Mueller EA, Steere AC, Palm NW, Grimes CL, Jacobs-wagner C. 2025. Bacterial and host enzymes modulate the inflammatory response produced by the peptidoglycan of the Lyme disease agent. bioRxiv [Preprint] 2025.01.08.631998.

46. Scheurwater E, Reid CW, Clarke AJ. 2008. Lytic transglycosylases: Bacterial space-making autolysins. Int J Biochem Cell Biol 40:586–591.

47. Dik DA, Marous DR, Fisher JF, Mobashery S. 2017. Lytic transglycosylases: concinnity in concision of the bacterial cell wall. Crit Rev Biochem Mol Biol 52:503–542.

48. Bachert BA, Bozue JA. 2023. Peptidoglycan enzymes of *Francisella*: Roles in cell morphology and pathogenesis, and potential as therapeutic targets. Front Microbiol 13:1099312.

49. Ramirez Carbo CA, Faromiki OG, Nan B. 2024. A lytic transglycosylase connects bacterial focal adhesion complexes to the peptidoglycan cell wall. Elife 13:RP99273.

50. Cloud KA, Dillard JP. 2002. A lytic transglycosylase of *Neisseria gonorrhoeae* is involved in peptidoglycan-derived cytotoxin production. Infect Immun 70:2752–2757.

51. Cloud-Hansen KA, Hackett KT, Garcia DL, Dillard JP. 2008. *Neisseria gonorrhoeae* uses two lytic transglycosylases to produce cytotoxic peptidoglycan monomers. J Bacteriol 190:5989–5994.

52. Kohler PL, Cloud KA, Hackett KT, Beck ET, Dillard JP. 2005. Characterization of the role of LtgB,a putative lytic transglycosylase in *Neisseria gonorrhoeae*. Microbiology 151:3081–3088.

53. Jennings GT, Savino S, Marchetti E, Aricò B, Kast T, Baldi L, Ursinus A, Höltje JV, Nicholas RA, Rappuoli R, Grandi G. 2002. GNA33 from *Neisseria meningitidis* serogroup B encodes a membrane-bound lytic transglycosylase (MltA). Eur J Biochem 269:3722–3731.

54. Harris-Jones TN, Pérez Medina KM, Hackett KT, Schave MA, Klimowicz AK, Schaub RE, Dillard JP. 2023. Mutation of mltG increases peptidoglycan fragment release, cell size, and antibiotic susceptibility in *Neisseria gonorrhoeae*. J Bacteriol 205:e0027723.

55. Kohler PL, Hamilton HL, Cloud-Hansen K, Dillard JP. 2007. AtlA functions as a peptidoglycan lytic transglycosylase in the *Neisseria gonorrhoeae* type IV secretion system. J Bacteriol 189:5421–5428.

56. Li JZ, Gu Y-L, Zhang W, Cong S, Wang R-N, Ma Y-N, Jin Y, Wei H-L. 2025. Pseudomonas syringae lytic transglycosylase HrpH interacts with host ubiquitin ligase ATL2 to modulate plant immunity. Cell Rep 44:115145.

57. García-Ramos M, de la Mora J, Ballado T, Camarena L, Dreyfus G. 2021. Modulation of the enzymatic activity of the flagellar lytic transglycosylase SltF by rod components and the scaffolding protein FlgJ in *Rhodobacter sphaeroides*. J Bacteriol 203:e00372-21.

58. Weaver A, Alvarez L, Rosch K, Ahmed A, Wang G, Van Nieuwenhze M, Cava F, Dörr T. 2022. Lytic transglycosylases mitigate periplasmic crowding by degrading soluble cell wall turnover products. Elife 11:e73178.

59. Oliveira ACP, Ferreira RM, Ferro MIT, Ferro JA, Chandler M, Varani AM. 2018. Transposons and pathogenicity in *Xanthomonas*: Acquisition of murein lytic transglycosylases by Tn*Xax*1 enhances *Xanthomonas citri* subsp. *citri* 306 virulence and fitness. PeerJ 6:e6111.

60. de Oliveira ACP, Ferreira RM, Ferro MIT, Ferro JA, Zamuner C, Ferreira H, Varani AM. 2022. XAC4296 is a multifunctional and exclusive *Xanthomonadaceae* gene containing a fusion of lytic transglycosylase and epimerase domains. Microorganisms 10.

61. Chen W, Xiao H, Wang L, Wang X, Tan Z, Han Z, Li X, Yang F, Liu Z, Song J, Liu H, Cheng L. 2021. Structural changes in bacteriophage T7 upon receptor-induced genome ejection. Proc Natl Acad Sci U S A 118:e2102003118.

62. Papayova K, Bocanova L, Bauerova V, Bauer J, Halgasova N, Kajsikova M, Bukovska G. 2025. From sequence to function: Exploring biophysical properties of bacteriophage BFK20 lytic transglycosylase domain from the minor tail protein gp15. Biochim Biophys Acta - Proteins Proteomics 1873:141044.

63. Utsunomiya H, Saiki N, Kadoguchi H, Fukudome M, Hashimoto S, Ueda M, Takechi K, Takano H. 2021. Genes encoding lipid II flippase MurJ and peptidoglycan hydrolases are required for chloroplast division in the moss *Physcomitrella patens*. Plant Mol Biol 107:405–415.

64. Lee SD, Yang HL, Kim JS, Kim IS. 2024. *Microbacterium aquilitoris* sp. nov. and *Microbacterium gwkjiense* sp. nov., isolated from beach. Arch Microbiol 206:100.

65. Lee SD. 2013. Proposal of *Motilibacteraceae* fam. nov., with the description of *Motilibacter rhizosphaerae* sp. nov. Int J Syst Evol Microbiol 63:3818–3822.

66. Mahapatra S, Crick DC, McNeil MR, Brennan PJ. 2008. Unique structural features of the peptidoglycan of *Mycobacterium leprae*. J Bacteriol 190:655–661.

67. Hansen JM, Golchin SA, Veyrier FJ, Domenech P, Boneca IG, Azad AK, Rajaram MVS, Schlesinger LS, Divangahi M, Reed MB, Behr MA. 2014. N-glycolylated peptidoglycan contributes to the immunogenicity but not pathogenicity of *Mycobacterium tuberculosis*. J Infect Dis 209:1045–1054.

68. Raymond JB, Mahapatra S, Crick DC, Pavelka MS. 2005. Identification of the *namH* gene, encoding the hydroxylase responsible for the N-glycolylation of the mycobacterial peptidoglycan. J Biol Chem 280:326–333.

69. Silveiro C, Marques M, Olivença F, Pires D, Mortinho D, Nunes A, Pimentel M, Anes E, Catalão MJ. 2023. CRISPRi-mediated characterization of novel anti-tuberculosis targets: Mycobacterial peptidoglycan modifications promote beta-lactam resistance and intracellular survival. Front Cell Infect Microbiol 13:1089911.

70. Gateau O, Bordet C, Michel G. 1976. Etude de la formation de l’acide N-glycolylmuramique du peptidoglycane de *Nocardia asteroides*. Biochim Biophys Acta 421:395–405.

71. Gilmore ME, Bandyopadhyay D, Dean AM, Linnstaedt SD, Popham DL. 2004. Production of muramic δ-lactam in *Bacillus subtilis* spore peptidoglycan. J Bacteriol 186:80–89.

72. Patin D, Boniface A, Kovač A, Hervé M, Dementin S, Barreteau H, Mengin-Lecreulx D, Blanot D. 2010. Purification and biochemical characterization of Mur ligases from *Staphylococcus aureus*. Biochimie 92:1793–1800.

73. Mahapatra S, Crick DC, Brennan PJ. 2000. Comparison of the UDP-*N*-acetylmuramate:L-alanine ligase enzymes from *Mycobacterium tuberculosis* and *Mycobacterium leprae*. J Bacteriol 182:6827–6830.

74. Hesse L, Bostock J, Dementin S, Blanot D, Mengin-Lecreulx D, Chopra I. 2003. Functional and biochemical analysis of *Chlamydia trachomatis* MurC, an enzyme displaying UDP-*N*-acetylmuramate:amino acid ligase activity. J Bacteriol 185:6507–6512.

75. Emanuele JJ, Jin H, Jacobson BL, Chang CY, Einspahr HM, Villafranca JJ. 1996. Kinetic and crystallographic studies of *Escherichia coli* UDP-*N*-acetylmuramate:L-alanine ligase. Protein Sci 5:2566–2574.

76. Song Q, Wu H, Zhang P, Zhu H, Xie J, Liu J, Qiao J. 2024. The MarR family regulator RmaH mediates acid tolerance of *Lactococcus lactis* through regulating peptidoglycan modification genes. J Dairy Sci 107:10383–10395.

77. Linnett PE, Strominger JL. 1974. Amidation and cross linking of the enzymatically synthesized peptidoglycan of *Bacillus stearothermophilus*. J Biol Chem 249:2489–2496.

78. Maitra A, Nukala S, Dickman R, Martin LT, Munshi T, Gupta A, Shepherd AJ, Arnvig KB, Tabor AB, Keep NH, Bhakta S. 2021. Characterization of the MurT/GatD complex in *Mycobacterium tuberculosis* towards validating a novel anti-tubercular drug target. JAC-Antimicrobial Resist 3:dlab028.

79. Shaku MT, Um PK, Ocius KL, Apostolos AJ, Pires MM, Bishai WR, Kana BD. 2024. A modified BCG with depletion of enzymes associated with peptidoglycan amidation induces enhanced protection against tuberculosis in mice. Elife 13:e89157.

80. Shaku MT, Ocius KL, Apostolos AJ, Pires MM, VanNieuwenhze MS, Dhar N, Kana BD. 2023. Amidation of glutamate residues in mycobacterial peptidoglycan is essential for cell wall cross-linking. Front Cell Infect Microbiol 13:1205829.

81. Münch D, Roemer T, Lee SH, Engeser M, Sahl HG, Schneider T. 2012. Identification and in vitro analysis of the GatD/MurT enzyme-complex catalyzing lipid II amidation in *Staphylococcus aureus*. PLoS Pathog 8:e1002509.

82. Morlot C, Straume D, Peters K, Hegnar OA, Simon N, Villard AM, Contreras-Martel C, Leisico F, Breukink E, Gravier-Pelletier C, Le Corre L, Vollmer W, Pietrancosta N, Håvarstein LS, Zapun A. 2018. Structure of the essential peptidoglycan amidotransferase MurT/GatD complex from *Streptococcus pneumoniae*. Nat Commun 9:3180.

83. Zapun A, Philippe J, Abrahams KA, Signor L, Roper DI, Breukink E, Vernet T. 2013. In vitro reconstitution of peptidoglycan assembly from the Gram-positive pathogen *Streptococcus pneumoniae*. ACS Chem Biol 8:2688–2696.

84. Evtushenko LI, Dorofeeva L V., Dobrovolskaya TG, Streshinskaya GM, Subbotin SA, Tiedje JM. 2001. *Agreia bicolorata* gen. nov., sp. nov., to accommodate actinobacteria isolated from narrow reed grass infected by the nematode *Heteroanguina graminophila*. Int J Syst Evol Microbiol 51:2073–2079.

85. Zhou X, Nan Guo G, Qi Wang L, Lan Bai S, Hong Li Y. 2016. *Cnuibacter physcomitrellae* gen. Nov., sp. nov., A novel member of the family *Microbacteriaceae* isolated from the moss of *Physcomitrella patens*. Int J Syst Evol Microbiol 66:680–688.

86. Matsumoto A, Kasai H, Matsuo Y, Ōmura S, Shizuri Y, Takahashi Y. 2009. *Ilumatobacter fluminis* gen. nov., sp. nov., a novel actinobacterium isolated from the sediment of an estuary. J Gen Appl Microbiol 55:201–205.

87. Kämpfer P, Rekha PD, Schumann P, Arun AB, Young CC, Chen WM, Sridhar KR. 2011. *Microbacterium arthrosphaerae* sp. nov., isolated from the faeces of the pill millipede *Arthrosphaera magna* Attems. Int J Syst Evol Microbiol 61:1334–1337.

88. Yoon JH, Schumann P, Kang SJ, Lee CS, Lee SY, Oh TK. 2009. *Microbacterium insulae* sp. nov., isolated from soil. Int J Syst Evol Microbiol 59:1738–1742.

89. Schleifer KH, Plapp R, Kandler O. 1967. Identification of threo-3-hydroxyglutamic acid in the cell wall of *Microbacterium lacticum*. Biochem Biophys Res Commun 28:566–570.

90. Mondani L, Piette L, Christen R, Bachar D, Berthomieu C, Chapon V. 2013. *Microbacterium lemovicicum* sp. nov., a bacterium isolated from a natural uranium-rich soil. Int J Syst Evol Microbiol 63:2600–2606.

91. Rahi P, Kurli R, Pansare AN, Khairnar M, Jagtap S, Patel NB, Dastager SG, Lawson PA, Shouche YS. 2018. *Microbacterium telephonicum* sp. nov., isolated from the screen of a cellular phone. Int J Syst Evol Microbiol 68:1052–1058.

92. Karojet S, Kunz S, van Dongen JT. 2012. *Microbacterium yannicii* sp. nov., isolated from *Arabidopsis thaliana* roots. Int J Syst Evol Microbiol 62:822–826.

93. Fiedler F, Schleifer K, Kandler O. 1973. Amino acid sequence of the threonine-containing mureins of Coryneform bacteria. J Bacteriol 113:8–17.

94. Interschick E, Fiedler F, Schleifer K, Kandler O. 1970. Glycine amide a constituent of the murein of *Arthrobacter atrocyaneus*. Z Naturforsch 256:714–717.

95. Schubert K, Reiml D, Accolas JP, Fiedler F. 1993. A novel type of *meso*-diaminopimelic acid-based peptidoglycan and novel poly(erythritol phosphate) teichoic acids in cell walls of two coryneform isolates from the surface flora of French cooked cheeses. Arch Microbiol 160:222–228.

96. Katz W, Matsuhashi M, Dietrich CP, Strominger JL. 1967. Biosynthesis of the peptidoglycan of bacterial cell walls. IV. Incorporation of glycine in *Micrococcus lysodeikticus* . J Biol Chem 242:3207–3217.

97. Whitney JG, Grula EA. 1968. A major attachment site for D-serine in the cell wall mucopeptide of *Micrococcus lysodeikticus*. Biochim Biophys Acta 158:124–129.

98. Severin A, Kokeguchi S, Kato K. 1989. Chemical composition of *Eubacterium nodatum* cell wall peptidoglycan. Arch Microbiol 151:353–358.

99. Hirao T, Sato M, Shirahata A, Kamio Y. 2000. Covalent linkage of polyamines to peptidoglycan in *Anaerovibrio lipolytica*. J Bacteriol 182:1154–1157.

100. Hamana K, Saito T, Okada M, Sakamoto A, Hosoya R. 2002. Covalently linked polyamines in the cell wall peptidoglycan of the anaerobes *Selenomonas, Anaeromusa, Dendrosporobacter, Acidaminococcus* and *Anaerovibrio* belonging to the *Sporomusa* subbranch. J Gen Appl Microbiol 48:177–180.

101. Kamio Y, Terawaki Y, Izaki K. 1982. Biosynthesis of cadaverine-containing peptidoglycan in *Selenomonas ruminantium*. J Biol Chem 257:3326–3333.

102. Ojima SK, Amio YK. 2012. Molecular basis for the maintenance of envelope integrity in *Selenomonas ruminantium*: Cadaverine biosynthesis and covalent modification into the peptidoglycan play a major role. J Nutr Sci Vitaminol (Tokyo) 58:153–160.

103. Kamio Y, Nakamura K. 1987. Putrescine and cadaverine are constituents of peptidoglycan in *Veillonella alcalescens* and *Veillonella parvula*. J Bacteriol 169:2881–2884.

104. Pfanzagl B, Allmaier G, Schmid ER, De Pedro MA, Löffelhardt W. 1996. *N*-Acetylputrescine as a characteristic constituent of cyanelle peptidoglycan in *Glaucocystophyte algae*. J Bacteriol 178:6994–6997.

105. Pfanzagl B, Löffelhardt W. 1999. In vitro synthesis of peptidoglycan precursors modified with *N*- acetylputrescine by *Cyanophora paradoxa* cyanelle envelope membranes. J Bacteriol 181:2643–2647.

106. Daniel R, Errington J. 1993. DNA sequence of the murE-murD region of *Bacillus subtilis* 168. J Gen Microbiol 139:361–370.

107. Rossini N de O, Silva C, Dias MVB. 2023. The crystal structure of *Mycobacterium thermoresistibile* MurE ligase reveals the binding mode of the substrate m-diaminopimelate. J Struct Biol 215:107957.

108. Basavannacharya C, Moody PR, Munshi T, Cronin N, Keep NH, Bhakta S. 2010. Essential residues for the enzyme activity of ATP-dependent MurE ligase from *Mycobacterium tuberculosis*. Protein Cell 1:1011–1022.

109. Jung KH, Kim YG, Kim CM, Ha HJ, Lee CS, Lee JH, Park HH. 2021. Wide-open conformation of UDP-MurNc-tripeptide ligase revealed by the substrate-free structure of MurE from *Acinetobacter baumannii*. FEBS Lett 595:275–283.

110. Shirakawa KT, Sala FA, Miyachiro MM, Job V, Trindade DM, Dessen A. 2017. Architecture and genomic arrangement of the MurE–MurF bacterial cell wall biosynthesis complex. Proc Natl Acad Sci 120:e2219540120.

111. Patin D, Bostock J, Chopra I, Mengin-Lecreulx D, Blanot D. 2012. Biochemical characterisation of the chlamydial MurF ligase, and possible sequence of the chlamydial peptidoglycan pentapeptide stem. Arch Microbiol 194:505–512.

112. Mengin-Lecreulx D, Blanot D, Van Heijenoort J. 1994. Replacement of diaminopimelic acid by cystathionine or lanthionine in the peptidoglycan of *Escherichia coli*. J Bacteriol 176:4321–4327.

113. Auger G, Van Heijenoort J, Vederas JC, Blanot D. 1996. Effect of analogues of diaminopimelic acid on the *meso*-diaminopimelate- adding enzyme from *Escherichia coli*. FEBS Lett 391:171–174.

114. Ma C, Redfield RJ. 2000. Point mutations in a peptidoglycan biosynthesis gene cause competence induction in *Haemophilus influenzae*. J Bacteriol 182:3323–3330.

115. Gharbia SE, Shah HN. 1990. Identification of *Fusobacterium* species by the electrophoretic migration of glutamate dehydrogenase and 2-oxoglutarate reductase in relation to their DNA base composition and peptidoglycan dibasic amino acids. J Med Microbiol 33:183–188.

116. Azzolina BA, Yuan X, Anderson MS, El-Sherbeini M. 2001. The cell wall and cell division gene cluster in the mra operon of *Pseudomonas aeruginosa*: Cloning, production, and purification of active enzymes. Protein Expr Purif 21:393–400.

117. Bansal R, Haque MA, Hassan MI, Ethayathulla AS, Kaur P. 2020. Structural and conformational behavior of MurE ligase from *Salmonella enterica* serovar Typhi at different temperature and pH conditions. Int J Biol Macromol 150:389–399.

118. McGroty SE, Pattaniyil DT, Patin D, Blanot D, Ravichandran AC, Suzuki H, Dobson RCJ, Savka MA, Hudson AO. 2013. Biochemical characterization of UDP-N-acetylmuramoyl-L-alanyl-D-glutamate: meso-2,6-diaminopimelate ligase (MurE) from *Verrucomicrobium spinosum* DSM 4136T. PLoS One 8:e66458.

119. Lin X, Li N, Kudo H, Zhang Z, Li J, Wang L, Zhang W, Takechi K, Takano H. 2017. Genes sufficient for synthesizing peptidoglycan are retained in gymnosperm genomes, and MurE from *Larix gmelinii* can rescue the albino phenotype of Arabidopsis MurE mutation. Plant Cell Physiol 58:587–597.

120. Garcia M, Myouga F, Takechi K, Sato H, Nabeshima K, Nagata N, Takio S, Shinozaki K, Takano H. 2008. An *Arabidopsis* homolog of the bacterial peptidoglycan synthesis enzyme MurE has an essential role in chloroplast development. Plant J 53:924–934.

121. Dowson AJ, Lloyd AJ, Cuming AC, Roper DI, Frigerio L, Dowson CG. 2022. Plant peptidoglycan precursor biosynthesis: Conservation between moss chloroplasts and Gram-negative bacteria. Plant Physiol 190:165–179.

122. Machida M, Takechi K, Sato H, Chung SJ, Kuroiwa H, Takio S, Seki M, Shinozaki K, Fujita T, Hasebe M, Takano H. 2006. Genes for the peptidoglycan synthesis pathway are essential for chloroplast division in moss. Proc Natl Acad Sci U S A 103:6753–6758.

123. Wijayarathna CD, Wachi M, Nagai K. 2001. Isolation of *ftsI* and *murE* genes involved in peptidoglycan synthesis from *Corynebacterium glutamicum*. Appl Microbiol Biotechnol 55:466–470.

124. Boudewijn L, de Jonge M, Gage D, Handwerger S. 1996. Peptidoglycan composition of vancomycin-resistant *Enterococcus faecium*. Microb Drug Resist 2:225–229.

125. Ruane KM, Lloyd AJ, Fülöp V, Dowson CG, Barreteau H, Boniface A, Dementin S, Blanot D, Mengin-Lecreulx D, Gobec S, Dessen A, Roper DI. 2013. Specificity determinants for lysine incorporation in *Staphylococcus aureus* peptidoglycan as revealed by the structure of a MurE enzyme ternary complex. J Biol Chem 288:33439–33448.

126. Blewett AM, Lloyd AJ, Echalier A, Fülöp V, Dowson CG, Bugg TDH, Roper DI. 2004. Expression, purification, crystallization and preliminary characterization of uridine 5′-diphospho-N-acetylmuramoyl L-alanyl-D-glutamate:lysine ligase (MurE) from *Streptococcus pneumoniae* 110K/70. Acta Crystallogr Sect D Biol Crystallogr D60:359–361.

127. Patin D, Turk S, Barreteau H, Mainardi JL, Arthur M, Gobec S, Mengin-Lecreulx D, Blanot D. 2016. Unusual substrate specificity of the peptidoglycan MurE ligase from *Erysipelothrix rhusiopathiae*. Biochimie 121:209–218.

128. Miyamoto T, Katane M, Saitoh Y, Sekine M, Homma H. 2019. Elucidation of the D-lysine biosynthetic pathway in the hyperthermophile *Thermotoga maritima*. FEBS J 286:601–614.

129. Boniface A, Bouhss A, Mengin-Lecreulx D, Blanot D. 2006. The MurE synthetase from *Thermotoga maritima* is endowed with an unusual D-lysine adding activity. J Biol Chem 281:15680–15686.

130. Kim W, Kim M, Park W. 2023. Unlocking the mystery of lysine toxicity on *Microcystis aeruginosa*. J Hazard Mater 448:130932.

131. Gerritsen J, Fuentes S, Grievink W, van Niftrik L, Tindall BJ, Timmerman HM, Rijkers GT, Smidt H. 2014. Characterization of *Romboutsia ilealis* gen. nov., sp. nov., isolated from the gastro-intestinal tract of a rat, and proposal for the reclassification of five closely related members of the genus *Clostridium* into the genera Romboutsia . Int J Syst Evol Microbiol 64:1600–1616.

132. Vasstrand EN, Hofstad T, Endresen C, Jensen HB. 1979. Demonstration of lanthionine as a natural constituent of the peptidoglycan of *Fusobacterium nucleatum*. Infect Immun 25:775–780.

133. Cho E, Park SN, Lim YK, Shin Y, Paek J, Hwang CH, Chang YH, Kook JK. 2015. *Fusobacterium hwasookii* sp. nov., isolated from a human periodontitis lesion. Curr Microbiol 70:169–175.

134. Frederiksen Å, Vasstrand EN, Jensen HB. 1991. Peptidoglycan precursor from *Fusobacterium nucleatum* contains lanthionine. J Bacteriol 173:900–902.

135. Yanagihara Y, Kamisango K, Yasuda S, Kobayashi S, Mifuchi I, Azuma I, Yamamura Y, Johnson RC. 1984. Chemical compositions of cell walls and polysaccharide fractions of Spirochetes. Microbiol Immunol 28:535–544.

136. Putnik R, Zhou J, Irnov I, Garner E, Liu M, Bersch KL, Jacobs-wagner C, Leimkuhler Grimes C. 2024. Synthesis of a *Borrelia burgdorferi*-derived muropeptide standard fragment library. Molecules 29:3297.

137. Umemoto T, Ota T, Sagawa H, Kato K, Takada H, Tsujimoto M, Kawasaki A, Ogawa T, Harada K, Kotani S. 1981. Chemical and biological properties of a peptidoglycan isolated from *Treponema pallidum* kazan. Infect Immun 31:767–774.

138. Ben Hania W, Joseph M, Schumann P, Bunk B, Fiebig A, Spröer C, Klenk H-P, Fardeau M-L, Spring S. 2015. Complete genome sequence and description of *Salinispira pacifica* gen. Nov., sp. nov., a novel spirochaete isolated form a hypersaline microbial mat. Stand Genomic Sci 10:1–14.

139. Zhou J, Zhang S, Zhang G, Yang J, Lai XH, Pu J, Jin D, Lu S, Huang Y, Zhu W, Huang Y, Xu M, Lei W, Cheng Y, Liu L, Xu J. 2021. Characterization of isolates of members of the genus *Actinomyces* from *Marmota himalayana*: Description of *Actinomyces faecalis* sp. nov., *Actinomyces respiraculi* sp. nov., and *Actinomyces trachealis* sp. nov. Int J Syst Evol Microbiol 71:004875.

140. Zhu W, Yang J, Lu S, Lai XH, Jin D, Wang X, Pu J, Ren Z, Huang Y, Wu X, Zhang X, Xu J, Xu J. 2020. *Actinomyces qiguomingii* sp. nov., isolated from the *Pantholops hodgsonii*. Int J Syst Evol Microbiol 70:58–64.

141. Hammes WP, Neukam R, Kandler O. 1977. On the specificity of the uridine diphospho-N-acetylmuramyl-alanyl-D-glutamic acid: diamino acid ligase of *Bifidobacterium globosum*. Arch Microbiol 115:95–102.

142. Yamamura H, Hayashi T, Hamada M, Kohda T, Serisawa Y, Matsuyama-Serisawa K, Nakagawa Y, Otoguro M, Yanagida F, Tamura T, Hayakawa M. 2019. *Cellulomonas algicola* sp. nov., an actinobacterium isolated from a freshwater alga. Int J Syst Evol Microbiol 69:2723–2728.

143. Li Y-Q, Zhang H, Xiao M, Dong ZY, Zhang J-Y, Rao MPN, Li W-J. 2020. *Cellulomonas endophytica* sp. nov., isolated from *Gastrodia elata blume*. Int J Syst Evol Microbiol 70:3091–3095.

144. Han C, Zhang Y, Yu B, Shan Q, Zhao J, Shi H, Tian Y, Zhang Y, Zhu C, Xiang W. 2022. *Cellulomonas triticagri* sp. nov., isolated from the rhizosphere soil of wheat (*Triticum aestivum* L.). Arch Microbiol 204:449.

145. Li Y-R, Zhu Z-N, Li Y-Q, Xiao M, Han M-X, Wadaan MAM, Hozzein WN, An D Di, Li W-J. 2018. *Microbacterium halophytorum* sp. nov., a novel endophytic actinobacterium isolated from halophytes. Int J Syst Evol Microbiol 68:3928–3934.

146. Li C, Jin X, Yang F, Zhao J, Wang S, Sun Q, Li L, Liu L. 2023. *Microbacterium nymphoidis* sp. nov. and *Microbacterium festucae* sp. nov., two novel species with high plant-promoting potential isolated from wetland plants in China. Int J Syst Evol Microbiol 73:006121.

147. Zhang L, Jiao Y, Ling L, Wang H, Song W, Zhao T, Guo L, Xiang W, Zhao J, Wang X. 2021. *Microbacterium stercoris* sp. nov., an indole acetic acid-producing actinobacterium isolated from cow dung. Int J Syst Evol Microbiol 71:005099.

148. Gao L, Fang B-Z, Liu Y-H, Huang Y, Jiao J-Y, Li L, Antunes A, Li W-J. 2022. *Ornithinimicrobium sediminis* sp. nov., a novel actinobacterium isolated from a saline lake sediment. Arch Microbiol 204:277.

149. Fang X-M, Du H-J, Bai J-L, He W-N, Li J, Wang H, Su J, Liu H-Y, Zhang Y-Q, Yu L-Y. 2020. *Ornithinimicrobium cerasi* sp. nov., isolated from the fruit of *Cerasus pseudocerasus* and emended description of the genus *Ornithinimicrobium*. Int J Syst Evol Microbiol 70:1691–1697.

150. Guinand M, Ghuysen J-M, Schleifer KH, Kandler O. 1969. The peptidoglycan in walls of *Butyribacterium rettgeri*. Biochemistry 8:200–207.

151. Liu Y, Li N, Eom MK, Schumann P, Zhang X, Cao Y, Ge Y, Xiao M, Zhao J, Cheng C, Kim S-G. 2017. *Bacillus ciccensis* sp. Nov., isolated from maize (*Zea mays* L.) seeds. Int J Syst Evol Microbiol 67:4606–4611.

152. Yu L, Tang X, Wei S, Qiu Y, Xu X, Xu G, Wang Q, Yang Q. 2019. Two novel species of the family *Bacillaceae*: *Oceanobacillus piezotolerans* sp. nov. and *Bacillus piezotolerans* sp. nov., from deep-sea sediment samples of yap trench. Int J Syst Evol Microbiol 69:3022–3030.

153. Li X, Zhang S, Gan L, Cai C, Tian Y, Shi B. 2020. *Ornithinibacillus caprae* sp. nov., a moderate halophile isolated from the hides of a white goat. Arch Microbiol 202:1469–1476.

154. Li R, Yang L, Pukall R, Neumann-Schaal M, Mu C-G, Shi Y-J, Wang Y, Jiang G-Q, Zhou Y-G, Cai M, Yin M, Zhu W-Y, Tang S-K. 2021. *Alkalibacillus aidingensis* sp. nov., an bacterium isolated from Aiding lake in Xinjiang province, north-west China. Curr Microbiol 78:3307–3312.

155. Li J, Kudo C, Tonouchi A. 2018. Description of *Deinococcus populi* sp. nov. from the trunk surface of a Japanese aspen tree. Arch Microbiol 200:291–297.

156. Ten LN, Cho H, Cho Y-J, Jung HY. 2019. *Deinococcus terrigena* sp. nov., a novel member of the family *Deinococcaceae*. Antonie Van Leeuwenhoek 112:389–399.

157. Moya G, Yan Z-F, Chu D-H, Won K, Yang J-E, Wang Q-J, Kook M-C, Yi T-H. 2018. *Deinococcus hibisci* sp. nov., isolated from rhizosphere of *Hibiscus syriacus* l. (mugunghwa flower). Int J Syst Evol Microbiol 68:28–34.

158. Quintela JC, Pittenauer E, Allmaier G, Aran V, De Pedro MA. 1995. Structure of peptidoglycan from *Thermus thermophilus* HB8. J Bacteriol 177:4947–4962.

159. Rachmania MK, Ningsih F, Sari DCAF, Sakai Y, Yokota A, Yabe S, Kim S-G, Sjamsuridzal W. 2024. *Dictyobacter halimunensis* sp. nov., a new member of the phylum *Chloroflexota*, from forest soil in a geothermal area. Int J Syst Evol Microbiol 74:006600.

160. Cole JK, Gieler BA, Heisler DL, Palisoc MM, Williams AJ, Dohnalkova AC, Ming H, Yu TT, Dodsworth JA, Li W-J, Hedlund BP. 2013. *Kallotenue papyrolyticum* gen. nov., sp. nov., a cellulolytic and filamentous thermophile that represents a novel lineage (*Kallotenuales* ord. nov., *Kallotenuaceae* fam. nov.) within the class Chloroflexia. Int J Syst Evol Microbiol 63:4675–4682.

161. Yan B, Guo X, Liu M, Huang Y. 2020. *Ktedonosporobacter rubrisoli* gen. nov., sp. nov., a novel representative of the class *Ktedonobacteria*, isolated from red soil, and proposal of *Ktedonosporobacteraceae* fam. nov. Int J Syst Evol Microbiol 70:1015–1025.

162. Keppen OI, Ivanovsky RN, Streshinskaya GM, Lebedeva N V., Shashkov AS, Dmitrenok AS, Grouzdev DS. 2018. The cell wall of the filamentous anoxygenic phototrophic bacterium *Oscillochloris trichoides*. Microbiology 164:57–64.

163. Dajkovic A, Tesson B, Chauhan S, Courtin P, Keary R, Flores P, Marlière C, Filipe SR, Chapot-Chartier M-P, Carballido-Lopez R. 2017. Hydrolysis of peptidoglycan is modulated by amidation of *meso*-diaminopimelic acid and Mg^2+^ in *Bacillus subtilis*. Mol Microbiol 104:972–988.

164. Ammam F, Patin D, Coullon H, Blanot D, Lambert T, Mengin-Lecreulx D, Candela T. 2020. AsnB is responsible for peptidoglycan precursor amidation in *Costridium difficile* in the presence of vancomycin. Microbiology 166:567–578.

165. Levefaudes M, Patin D, de Sousa-D’Auria C, Chami M, Blanot D, Hervé M, Arthur M, Houssin C, Mengin-Lecreulx D. 2015. Diaminopimelic acid amidation in Corynebacteriales. New insights into the role of LtsA in peptidoglycan modification. J Biol Chem 290:13079–13094.

166. Bernard E, Rolain T, Courtin P, Hols P, Chapot-Chartier MP. 2011. Identification of the amidotransferase AsnB1 as being responsible for *meso*-diaminopimelic acid amidation in *Lactobacillus plantarum* peptidoglycan. J Bacteriol 193:6323–6330.

167. Sun L, Rogiers G, Courtin P, Chapot-Chartier M-P, Bierne H, Michiels CW. 2021. AsnB mediates amidation of *meso*-diaminopimelic acid residues in the peptidoglycan of *Listeria monocytogenes* and affects bacterial surface properties and host cell invasion. Front Microbiol 12:760253.

168. Ngadjeua F, Braud E, Saidjalolov S, Iannazzo L, Schnappinger D, Ehrt S, Hugonnet J-E, Mengin-Lecreulx D, Patin D, Ethève-Quelquejeu M, Fonvielle M, Arthur M. 2018. Critical impact of peptidoglycan precursor amidation on the activity of L,D-transpeptidases from *Enterococcus faecium* and *Mycobacterium tuberculosis*. Chemistry (Easton) 24:5743–5747.

169. Trippen B, Hammes WP, Schleifer KH, Kandler O. 1976. Mode of action of D-amino acids on the biosynthesis of peptidoglycan. Arch Microbiol 109:247–261.

170. Lam H, Oh D-C, Cava F, Takacs CN, Jon C, de Pedro M a., Waldor MK. 2009. D-Amino acids govern stationary phase cell wall re-modeling in bacteria. Science (80- ) 325:1552–1555.

171. Baranowski C, Welsh MA, Sham LT, Eskandarian HA, Lim HC, Kieser KJ, Wagner JC, McKinney JD, Fantner GE, Ioerger TR, Walker S, Bernhardt TG, Rubin EJ, Rego EH. 2018. Maturing *Mycobacterium smegmatis* peptidoglycan requires non-canonical crosslinks to maintain shape. Elife 7:e37516.

172. Sibinelli-Sousa S, Hespanhol JT, Nicastro GG, Matsuyama BY, Mesnage S, Patel A, de Souza RF, Guzzo CR, Bayer-Santos E. 2020. A family of T6SS antibacterial effectors related to L,D-transpeptidases targets the peptidoglycan. Cell Rep 31:107813.

173. Lorente Cobo N, Sibinelli-Sousa S, Biboy J, Vollmer W, Bayer-Santos E, Prehna G. 2022. Molecular characterization of the type VI secretion system effector Tlde1a reveals a structurally altered LD-transpeptidase fold. J Biol Chem 298:102556.

174. Le NH, Peters K, Espaillat A, Sheldon JR, Gray J, Venanzio G Di, Lopez J, Djahanschiri B, Mueller EA, Hennon SW, Levin PA, Ebersberger I, Skaar EP, Cava F, Vollmer W, Feldman MF. 2020. Peptidoglycan editing provides immunity to *Acinetobacter baumannii* during bacterial warfare. Sci Adv 6:eabb5614 22.

175. Magnet S, Dubost L, Marie A, Arthur M, Gutmann L. 2008. Identification of the L,D-transpeptidases for peptidoglycan cross-linking in *Escherichia coli*. J Bacteriol 190:4782–4785.

176. Kuru E, Radkov A, Meng X, Egan A, Alvarez L, Dowson A, Booher G, Breukink E, Roper DI, Cava F, Vollmer W, Brun Y, Vannieuwenhze MS. 2019. Mechanisms of incorporation for D-amino acid probes that target peptidoglycan biosynthesis. ACS Chem Biol 14:2745–2756.

177. Cava F, De Pedro MA, Lam H, Davis BM, Waldor MK. 2011. Distinct pathways for modification of the bacterial cell wall by non-canonical D-amino acids. EMBO J 30:3442–3453.

178. Espaillat A, Carrasco-López C, Bernardo-García N, Rojas-Altuve A, Klett J, Morreale A, Hermoso JA, Cava F. 2021. Binding of non-canonical peptidoglycan controls *Vibrio cholerae* broad spectrum racemase activity. Comput Struct Biotechnol J 19:1119–1126.

179. Hernández SB, Castanheira S, Graciela Pucciarelli M, Cestero JJ, Rico-Pérez G, Paradela A, Ayala JA, Velázquez S, San-Félix A, Cava F, García-del Portillo F. 2022. Peptidoglycan editing in non-proliferating intracellular *Salmonella* as source of interference with immune signaling. PLoS Pathog 18:e1010241.

180. Mahapatra S, Yagi T, Belisle JT, Espinosa BJ, Hill PJ, McNeil MR, Brennan PJ, Crick DC. 2005. Mycobacterial lipid II is composed of a complex mixture of modified muramyl and peptide moieties linked to decaprenyl phosphate. J Bacteriol 187:2747–2757.

181. Anderson EM, Shaji Saji N, Anderson AC, Brewer D, Clarke AJ, Khursigara CM. 2022. *Pseudomonas aeruginosa* alters peptidoglycan composition under nutrient conditions resembling cystic fibrosis lung infections. mSystems 7:e0015622.

182. Leiman SA, May JM, Lebar MD, Kahne D, Kolter R, Losick R. 2013. D-Amino acids indirectly inhibit biofilm formation in *Bacillus subtilis* by interfering with protein synthesis. J Bacteriol 195:5391–5395.

183. Qiao Y, Lebar MD, Schirner K, Schaefer K, Tsukamoto H, Kahne D, Walker S. 2014. Detection of lipid-linked peptidoglycan precursors by exploiting an unexpected transpeptidase reaction. J Am Chem Soc 136:14678–14681.

184. Lupoli TJ, Tsukamoto H, Doud EH, Wang T-SA, Walker S, Kahne D. 2011. Transpeptidase-mediated incorporation of D-amino acids into bacterial peptidoglycan. J Am Chem Soc 133:10748–10751.

185. Meziane-Cherif D, Badet-Denisot M-A, Evers S, Courvalin P, Badet B. 1994. Purification and characterization of the VanB ligase associated with type B vancomycin resistance in *Enterococcus faecalis* V583. FEBS Lett 354:140–142.

186. Depardieu F, Kolbert M, Pruul H, Bell J, Courvalin P. 2004. VanD-type vancomycin-resistant *Enterococcus faecium* and *Enterococcus faecalis*. Antimicrob Agents Chemother 48:3892–3904.

187. Bugg TD, Wright GD, Dutka-Malen S, Arthur M, Courvalin P, Walsh CT. 1991. Molecular basis for vancomycin resistance in *Enterococcus faecium* BM4147: biosynthesis of a depsipeptide peptidoglycan precursor by vancomycin resistance proteins VanH and VanA. Biochemistry 30:10408–10415.

188. Xu X, Lin D, Yan G, Ye X, Wu S, Guo Y, Zhu D, Hu F, Zhang Y, Wang F, Jacoby GA, Wang M. 2010. *vanM*, a new glycopeptide resistance gene cluster found in *Enterococcus faecium*. Antimicrob Agents Chemother 54:4643–4647.

189. Song X, Li F, Zhang M, Xia Y, Ai L, Wang G. 2022. Effect of D-Ala-ended peptidoglycan precursors on the immune regulation of *Lactobacillus plantarum* strains. Front Immunol 12:825825.

190. Périchon B, Courvalin P. 2009. VanA-type vancomycin-resistant *Staphylococcus aureus*. Antimicrob Agents Chemother 53:4580–4587.

191. Boudrioua A, Li Y, Hartke A, Giraud C. 2020. Opposite effect of vancomycin and D-cycloserine combination in both vancomycin resistant *Staphylococcus aureus* and *enterococci*. FEMS Microbiol Lett 367:fnaa062.

192. Foucault M-L, Courvalin P, Grillot-Courvalin C. 2009. Fitness cost of VanA-type vancomycin resistance in methicillin-resistant *Staphylococcus aureus*. Antimicrob Agents Chemother 53:2354–2359.

193. Hong H-J, Hutchings MI, Neu JM, Wright GD, Paget MSB, Buttner MJ. 2004. Characterization of an inducible vancomycin resistance system in *Streptomyces coelicolor* reveals a novel gene (*vanK*) required for drug resistance. Mol Microbiol 52:1107–1121.

194. Marshall CG, Broadhead G, Leskiw BK, Wright GD. 1997. D-Ala-D-Ala ligases from glycopeptide antibiotic-producing organisms are highly homologous to the enterococcal vancomycin-resistance ligases VanA and VanB. Proc Natl Acad Sci U S A 94:6480–6483.

195. Marshall CG, Lessard IAD, Park IS, Wright GD. 1998. Glycopeptide antibiotic resistance genes in glycopeptide-producing organisms. Antimicrob Agents Chemother 42:2215–2220.

196. Ammam F, Meziane-cherif D, Mengin-Lecreulx D, Blanot D, Patin D, Boneca IG, Courvalin P, Lambert T, Candela T. 2013. The functional vanG_Cd_ cluster of *Clostridium difficile* does not confer vancomycin resistance. Mol Microbiol 89:612–625.

197. Reynolds PE, Snaith HA, Maguire AJ, Dutka-Malen S, Courvalin P. 1994. Analysis of peptidoglycan precursors in vancomycin-resistant *Enterococcus gallinarum* BM4174. Biochem J 301:5–8.

198. Arias CA, Courvalin P, Reynolds PE. 2000. *vanC* Cluster of vancomycin-resistant *Enterococcus gallinarum* BM4174. Antimicrob Agents Chemother 44:1660–1666.

199. Watanabe S, Kobayashi N, Quiñones D, Hayakawa S, Nagashima S, Uehara N, Watanabe N. 2009. Genetic diversity of the low-level vancomycin resistance gene vanC-2/vanC-3 and identification of a novel vanC subtype (vanC-4) in *Enterococcus casseliflavus* . Microb Drug Resist 15.

200. Park IS, Lin C-H, Walsh CT. 1997. Bacterial resistance to vancomycin: Overproduction, purification, and characterization of VanC2 from *Enterococcus casseliflavus* as a D-Ala-D-Ser ligase. Proc Natl Acad Sci U S A 94:10040–10044.

201. Navarro F, Courvalin P. 1994. Analysis of genes encoding D-alanine-D-alanine ligase-related enzymes in *Enterococcus casseliflavus* and *Enterococcus flavescens*. Antimicrob Agents Chemother 38:1788–1793.

202. Hachem Y, Djouadi LN, Raddaoui A, Boukli-Hacene F, Boumerdassi H, Achour W, Nateche F. 2024. Phenotypic and molecular characterization of vancomycin resistant enterococci from wild birds: first detection of a plasmid-borne vanC1 in *Enterococcus faecalis*. Lett Appl Microbiol 77:ovae098.

203. Weber P, Meziane-Cherif D, Haouz A, Saul FA, Courvalin P. 2009. Crystallization and preliminary X-ray analysis of a D-Ala:D-Ser ligase associated with VanG-type vancomycin resistance. Acta Crystallogr Sect F Struct Biol Cryst Commun 65:1024–1026.

204. Depardieu F, Bonora MG, Reynolds PE, Courvalin P. 2003. The *vanG* glycopeptide resistance operon from *Enterococcus faecalis* revisited. Mol Microbiol 50:931–948.

205. Fines M, Perichon B, Reynolds P, Sahm DF, Courvalin P. 1999. VanE, a new type of acquired glycopeptide resistance in *Enterococcus faecalis* BM4405. Antimicrob Agents Chemother 43:2161–2164.

206. Abadía-Patiño L, Christiansen K, Bell J, Courvalin P, Périchon B. 2004. VanE-type vancomycin-resistant *Enterococcus faecalis* clinical isolates from Australia. Antimicrob Agents Chemother 48:4882–4885.

207. Boyd DA, Willey BM, Fawcett D, Gillani N, Mulvey MR. 2008. Molecular characterization of *Enterococcus faecalis* N06-0364 with low-level vancomycin resistance harboring a novel D-Ala-D-Ser gene cluster, *vanL*. Antimicrob Agents Chemother 52:2667–2672.

208. Lebreton F, Depardieu F, Bourdon N, Fines-Guyon M, Berger P, Camiade S, Leclercq R, Courvalin P, Cattoir V. 2011. D-Ala-D-Ser VanN-type transferable vancomycin resistance in *Enterococcus faecium*. Antimicrob Agents Chemother 55:4606–4612.

209. Wu H, Xue E, Zhi N, Song Q, Tian K, Caiyin Q, Yuan L, Qiao J. 2020. D-Methionine and D-phenylalanine improve *Lactococcus lactis* F44 acid resistance and nisin yield by governing cell wall remodeling. Appl Environ Microbiol 86:e02981-19.

210. Meyer FM, Bramkamp M. 2024. Cell wall synthesizing complexes in *Mycobacteriales*. Curr Opin Microbiol 79:102478.

211. Pinho MG, Foster SJ. 2024. Cell growth and division of *Staphylococcus aureus*. Annu Rev Microbiol 78:293–310.

212. Vollmer W, Massidda O, Tomasz A. 2019. The cell wall of *Streptococcus pneumoniae*. Microbiol Spectr 7:GPP3-0018–2018.

213. Dhar S, Kumari H, Balasubramanian D, Mathee K. 2018. Cell-wall recycling and synthesis in *Escherichia coli* and *Pseudomonas aeruginosa* – Their role in the development of resistance. J Med Microbiol 67:1–21.

214. Egan AJF, Errington J, Vollmer W. 2020. Regulation of peptidoglycan synthesis and remodelling. Nat Rev Microbiol 18:446–460.

215. Kwan JMC, Qiao Y. 2023. Mechanistic insights into the activities of major families of enzymes in bacterial peptidoglycan assembly and breakdown. Chembiochem A Eur J Chem Biol 24:e202200693.

216. Henrichfreise B, Brunke M, Viollier PH. 2016. Bacterial Surfaces: The Wall that SEDS Built. Curr Biol 26:R1158–R1160.

217. Galinier A, Delan-Forino C, Foulquier E, Lakhal H, Pompeo F. 2023. Recent advances in peptidoglycan synthesis and regulation in bacteria. Biomolecules 13:720.

218. Peltier J, Courtin P, El Meouche I, Lemée L, Chapot-Chartier MP, Pons J-L. 2011. *Clostridium difficile* has an original peptidoglycan structure with a high level of *N*-acetylglucosamine deacetylation and mainly 3-3 cross-links. J Biol Chem 286:29053–29062.

219. Bollinger KW, Müh U, Ocius KL, Apostolos AJ, Pires MM, Helm RF, Popham DL, Weiss DS, Ellermeier CD. 2024. Identification of a family of peptidoglycan transpeptidases reveals that *Clostridioides difficile* requires noncanonical cross- links for viability. Proc Natl Acad Sci 121:e2408540121.

220. Mainardi J-L, Fourgeaud M, Hugonnet J-E, Dubost L, Brouard J-P, Ouazzani J, Rice LB, Gutmann L, Arthur M. 2005. A novel peptidoglycan cross-linking enzyme for a β-lactam-resistant transpeptidation pathway. J Biol Chem 280:38146–38152.

221. Lavollay M, Arthur M, Fourgeaud M, Dubost L, Marie A, Veziris N, Blanot D, Gutmann L, Mainardi J-L. 2008. The peptidoglycan of stationary-phase *Mycobacterium tuberculosis* predominantly contains cross-links generated by L,D-transpeptidation. J Bacteriol 190:4360–4366.

222. de Munnik M, Lang PA, Calvopiña K, Rabe P, Brem J, Schofield CJ. 2024. Biochemical and crystallographic studies of L,D-transpeptidase 2 from *Mycobacterium tuberculosis* with its natural monomer substrate. Commun Biol 7:1173.

223. Libreros-Zúniga GA, Dos Santos Silva C, Ferreira RS, Dias MVB. 2019. Structural basis for the interaction and processing of β-lactam antibiotics by L,D-transpeptidase 3 (Ldt Mt3 ) from *Mycobacterium tuberculosis*. ACS Infect Dis 5:260–271.

224. Toth M, Stewart NK, Smith CA, Lee M, Vakulenko SB. 2022. The L,D-transpeptidase Ldt_Ab_ from *Acinetobacter baumannii* is poorly inhibited by carbapenems and has a unique structural architecture. ACS Infect Dis 8:1948–1961.

225. Aliashkevich A, Guest T, Alvarez L, Gilmore MC, Rea D, Amstutz J, Mateus A, Schiffthaler B, Ruiz I, Typas A, Savitski MM, Brown PJB, Cava F. 2024. LD-transpeptidation is crucial for fitness and polar growth in *Agrobacterium tumefaciens*. PLoS Genet 20:e1011449.

226. Kathayat D, Huang Y, Denis J, Rudoy B, Schwarz H, Szlechter J, Kathayat D, Huang Y, Denis J, Rudoy B, Schwarz H, Szlechter J. 2025. LD-transpeptidase-mediated cell envelope remodeling enables developmental transitions and survival in *Coxiella burnetii* and *Legionella pneumophila*. J Bacteriol 207:e0024724.

227. Hugonneau-Beaufet I, Barnier J-P, Thiriet-Rupert S, Létoffé S, Mainardi J-L, Ghigo J-M, Beloin C, Arthur M. 2023. Characterization of *Pseudomonas aeruginosa* L,D -transpeptidases and evaluation of their role in peptidoglycan adaptation to biofilm growth. Microbiol Spectr 11:e0521722.

228. Espaillat A, Alvarez L, Torrens G, ter Beek J, Miguel-Ruano V, Irazoki O, Gago F, Hermoso JA, Berntsson RPA, Cava F. 2024. A distinctive family of L,D-transpeptidases catalyzing L-Ala-*m*DAP crosslinks in Alpha- and Betaproteobacteria. Nat Commun 15:1343.

229. Bouhss A, Josseaume N, Severin A, Tabei K, Hugonnet JE, Shlaes D, Mengin-Lecreulx D, van Heijenoort J, Arthur M. 2002. Synthesis of the L-alanyl-L-alanine cross-bridge of *Enterococcus faecalis* peptidoglycan. J Biol Chem 277:45935–45941.

230. Bellais S, Arthur M, Dubost L, Hugonnet J-E, Gutmann L, van Heijenoort J, Legrand R, Brouard J-P, Rice L, Mainardi J-L. 2006. Asl_fm_, the D-aspartate ligase responsible for the addition of D-aspartic acid onto the peptidoglycan precursor of *Enterococcus faecium*. J Biol Chem 281:11586–11594.

231. Veiga P, Piquet S, Maisons A, Furlan S, Courtin P, Chapot-Chartier M-P, Kulakauskas S. 2006. Identification of an essential gene responsible for D-Asp incorporation in the *Lactococcus lactis* peptidoglycan crossbridge. Mol Microbiol 62:1713–1724.

232. Strandén AM, Ehlert K, Labischinski H, Berger-Bӓchi B. 1997. Cell wall monoglycine cross-bridges and methicillin hypersusceptibility in a *femAB* null mutant of methicillin-resistant *Staphylococcus aureus*. J Bacteriol 179:9–16.

233. Rohrer S, Ehlert K, Tschierske M, Labischinski H, Berger-Bӓchi B. 1999. The essential *Staphylococcus aureus* gene *fmhB* is involved in the first step of peptidoglycan pentaglycine interpeptide formation. Proc Natl Acad Sci U S A 96:9351–9356.

234. Schneider T, Senn MM, Berger-Bächi B, Tossi A, Sahl H-G, Wiedemann I. 2004. In vitro assembly of a complete, pentaglycine interpeptide bridge containing cell wall precursor (lipid II-Gly5) of *Staphylococcus aureus*. Mol Microbiol 53:675–685.

235. Willing S, Dyer E, Schneewind O, Missiakas D. 2020. FmhA and FmhC of *Staphylococcus aureus* incorporate serine residues into peptidoglycan cross-bridges. J Biol Chem 295:13664–13676.

236. Todorova K, Maurer P, Rieger M, Becker T, Bui NK, Gray J, Vollmer W, Hakenbeck R. 2015. Transfer of penicillin resistance from *Streptococcus oralis* to *Streptococcus pneumoniae* identifies *murE* as resistance determinant. Mol Microbiol 97:866–880.

237. Filipe SR, Pinho MG, Tomasz A. 2000. Characterization of the *murMN* operon involved in the synthesis of branched peptidoglycan peptides in *Streptococcus pneumoniae*. J Biol Chem 275:27768–27774.

238. Gargis SR, Gargis AS, Heath HE, Heath LS, LeBlanc PA, Senn MM, Berger-Bächi B, Simmonds RS, Sloan GL. 2009. Zif, the zoocin A immunity factor, is a FemABX-like immunity protein with a novel mode of action. Appl Environ Microbiol 75:6205–6210.

239. Hong H-J, Hutchings MI, Hill LM, Buttner MJ. 2005. The role of the novel fem protein VanK in vancomycin resistance in *Streptomyces coelicolor*. J Biol Chem 280:13055–13061.

240. Maillard AP, Biarrotte-Sorin S, Villet R, Mesnage S, Bouhss A, Sougakoff W, Mayer C, Arthur M. 2005. Structure-based site-directed mutagenesis of the UDP-MurNAc-pentapeptide- binding cavity of the FemX alanyl transferase from *Weissella viridescens*. J Bacteriol 187:3833–3838.
